# Global prevalence of preeclampsia, eclampsia, and HELLP syndrome: a systematic review and meta-analysis

**DOI:** 10.3389/frph.2025.1706009

**Published:** 2025-11-10

**Authors:** Víctor Juan Vera-Ponce, Joan A. Loayza-Castro, Jhosmer Ballena-Caicedo, Lupita Ana Maria Valladolid-Sandoval, Fiorella E. Zuzunaga-Montoya, Carmen Inés Gutierrez De Carrillo

**Affiliations:** 1 Instituto de Investigación de Enfermedades Tropicales, Universidad Nacional Toribio Rodríguez de Mendoza de Amazonas (UNTRM), Amazonas, Perú; 2 Facultad de Medicina (FAMED), Universidad Nacional Toribio Rodríguez de Mendoza de Amazonas (UNTRM), Amazonas, Perú; 3 Universidad Continental, Lima, Perú

**Keywords:** preeclampsia, eclampsia, HELLP syndrome, prevalence, epidemiology, meta-analysis, hypertension, pregnancy-induced, maternal health

## Abstract

**Introduction:**

Hypertensive disorders of pregnancy represent a leading cause of maternal and perinatal morbidity and mortality worldwide. However, prevalence estimates of preeclampsia, eclampsia, and HELLP syndrome vary considerably across studies and regions.

**Objective:**

To determine the global prevalence of preeclampsia, eclampsia, and HELLP (Hemolysis, Elevated Liver enzymes, and Low Platelet count) syndrome, analyze their geographical distribution, and evaluate temporal and methodological trends.

**Methodology:**

A systematic review with meta-analysis was conducted. SCOPUS, Web of Science, PubMed, and EMBASE databases were searched through May 2025. Observational studies reporting prevalence data using standardized diagnostic criteria were included. Prevalences were pooled using a random-effects model with Freeman-Tukey double arcsine transformation. Subgroup analyses by diagnostic criteria and countries and meta-regressions by publication year and sample size were performed.

**Results:**

Seventy studies on preeclampsia (2,465,570 participants), 21 on eclampsia (9,782,257 participants), and nine on HELLP syndrome (133,611 participants) were analyzed. The global prevalence of preeclampsia was 4.43 (95% CI: 3.73–5.20), with significant differences between ACOG (4.68%) and ISSHP (3.66%) criteria. For eclampsia, the prevalence was 0.43% (95% CI: 0.19%–0.76%), while the estimate for HELLP syndrome is 0.39% (95% CI: 0.16%–0.72%), which must be interpreted with considerable caution as it is derived from a limited pool of only nine studies. Marked regional disparities were identified, with higher prevalences in low-income countries. Meta-regression for preeclampsia revealed a non-significant increasing trend over time (*p* = 0.23) and a significant inverse correlation with sample size (*p* < 0.01). For eclampsia, neither the temporal trend (*p* = 0.68) nor the association with sample size (*p* = 0.65) was statistically significant.

**Conclusions:**

Hypertensive disorders of pregnancy affect 4.43% (95% CI: 3.73%–5.20%) of pregnancies globally for preeclampsia, 0.43% (95% CI: 0.19%–0.76%) for eclampsia, and 0.39% (95% CI: 0.16%–0.72%) for HELLP syndrome, with considerable variations according to regions and diagnostic criteria. The upward trend underscores the need to strengthen epidemiological surveillance systems and preventive programs, especially in high-prevalence areas.

## Introduction

Preeclampsia is a hypertensive disorder that occurs during pregnancy, characterized by the onset of hypertension and signs of end-organ damage after 20 weeks of gestation in previously normotensive women (1). It is estimated to affect between 2% and 8% of all pregnancies worldwide, making it one of the leading causes of maternal and perinatal morbidity and mortality (2).

The global burden of preeclampsia is not uniform, with its prevalence varying significantly due to an interplay between population-level risk factors and health system capacity (2). Epidemiological data show that in low- and middle-income countries, limited access to high-quality prenatal care can delay diagnosis, increasing the risk of severe complications (3). Concurrently, demographic trends such as advanced maternal age and a rising prevalence of obesity, alongside comorbidities like chronic hypertension and diabetes, are established risk factors that contribute to the increasing incidence of this condition globally (4, 5). The heterogeneous distribution of these risk profiles and the disparities in healthcare infrastructure are fundamental drivers of the variations observed in prevalence rates across different regions.

The clinical consequences of preeclampsia extend far beyond the gestational period, posing significant long-term health risks. For the mother, a history of preeclampsia is a potent risk factor for future cardiovascular events, including chronic hypertension, ischemic heart disease, and stroke. For the offspring, exposure to preeclampsia *in utero* has been associated with adverse neurological and metabolic sequelae later in life (3, 6, 7). In the short term, progression to eclampsia or the development of HELLP syndrome represents an immediate threat to both maternal and fetal life. Therefore, prevention, timely diagnosis, and appropriate management remain priority objectives in obstetric care (1).

In this context, having precise and updated estimates of preeclampsia's global prevalence is essential for designing effective public health interventions. While previous systematic reviews have provided foundational global estimates, rapid shifts in risk factor prevalence and diagnostic practices necessitate an updated synthesis. Significant heterogeneity in published data persists, and a comprehensive analysis examining how methodological factors influence prevalence has been lacking. In particular, the divergence between major international guidelines, such as those from the American College of Obstetricians and Gynecologists (ACOG) (8) and the International Society for the Study of Hypertension in Pregnancy (ISSHP) (9), contributes significantly to this heterogeneity. Hence, this systematic review and meta-analysis aims to synthesize published data, identify knowledge gaps, and guide health policymakers with robust, evidence-based insights.

## Methodology

### Study design

This work was conceived as a systematic review with a meta-analysis of studies that evaluated the prevalence of preeclampsia, eclampsia, or HELLP syndrome in different geographical and population contexts. For the development of the protocol and subsequent execution of the review, the guidelines of the PRISMA Statement (Preferred Reporting Items for Systematic Reviews and Meta-Analyses) were followed, making relevant adaptations as this is a prevalence review (10). In this regard, specific methodological guidelines recommended for systematic reviews of observational studies reporting prevalence data were considered, such as those proposed by Munn et al. (11).

### Search strategy

Following the methodological recommendations of the Cochrane Collaboration for systematic reviews (12), a search strategy was designed to identify studies reporting the prevalence of the diseases above through May 2025. To this end, the databases SCOPUS, Web of Science (WOS, including the SciELO catalog), PubMed, and EMBASE were consulted, selected for their broad coverage of scientific literature, and for being suggested sources in these guidelines for high-quality systematic reviews.

To cover the topic of interest, the main keywords “Preeclampsia,” “Eclampsia,” or “HELLP,” and “prevalence” were used, combining them with Boolean operators (AND, OR) as appropriate. When applicable, both free terms and controlled terms (e.g., MeSH in PubMed and Emtree in EMBASE) were employed to maximize the retrieval of relevant studies. The detailed search strategy, including specific equations and applied limits, is presented in Supplementary Material 1.

### Selection criteria

Observational studies that provided specific data on the events’ prevalence were included, regardless of whether their samples were selected using probabilistic or non-probabilistic methods. Both cross-sectional and cohort studies were considered eligible, provided they supplied clear epidemiological information on the condition's prevalence at the time of evaluation.

To ensure the validity and comparability of data, selected studies were required to employ standardized and internationally recognized diagnostic criteria for preeclampsia, eclampsia, and HELLP syndrome. ACOG criteria (8) define preeclampsia as systolic blood pressure ≥140 mmHg or diastolic blood pressure ≥90 mmHg on two occasions at least 4 h apart after 20 weeks of gestation in previously normotensive women, accompanied by proteinuria (≥300 mg per 24-h urine collection) or, in the absence of proteinuria, new-onset hypertension with severe features including thrombocytopenia, impaired liver function, renal insufficiency, pulmonary edema, or cerebral/visual disturbances. ISSHP criteria (9) similarly define preeclampsia as *de novo* hypertension (≥140/90 mmHg) arising after 20 weeks of gestation combined with proteinuria, maternal organ dysfunction, or uteroplacental dysfunction. Eclampsia was defined as the occurrence of seizures that cannot be attributed to other causes in women with preeclampsia. HELLP syndrome was characterized by the triad of hemolysis, elevated liver enzymes, and low platelet count, with specific laboratory thresholds defined according to the respective classification systems employed by each study. Studies using ICD-9 or ICD-10 codes were included when these corresponded to the clinical definitions described above (13). No restrictions were imposed regarding language or publication date, provided the articles presented quantifiable and methodologically appropriate information on the prevalence of preeclampsia.

### Study selection process

After completing the bibliographic search in the selected databases, all identified records were imported into the Rayyan platform, an online tool that facilitates the articles’ screening and selection process. Two reviewers (JJBC and LAMVS) independently evaluated titles and abstracts with Rayyan's blinding feature active. Blinding was removed only after both reviewers had completed this initial screening phase, allowing for the comparison of decisions and the identification of discrepancies.

When disagreements about the inclusion or exclusion of a study arose, they were first resolved through discussion to reach a consensus. In cases where a consensus could not be reached, a third researcher (VJVP) was consulted to issue the definitive ruling. This systematic procedure ensured a comprehensive and transparent literature review, thus reducing the risk of selection bias.

### Data extraction and qualitative analysis

Following the selection process, articles that met the inclusion criteria were entered into a template designed in Microsoft Excel 2023. Two reviewers (VJVP and JALC) independently extracted relevant information from each study, using a standardized recording sheet to ensure the collected data's consistency and comprehensiveness. In case of discrepancies between reviewers, a joint discussion was held until consensus was achieved. If consensus could not be reached, a third reviewer (CIGDC) provided the final resolution.

The extracted data encompassed details on the methodological characteristics and results of each investigation, including author(s) and year of publication, Latin American country or countries contemplated, type of study and data collection period, sample size, and demographic characteristics (e.g., population age), sampling method employed, diagnostic criteria used for preeclampsia, as well as the reported prevalence and main findings related to the variable of interest. Based on this information, a descriptive qualitative analysis of the characteristics of the selected studies was conducted to identify patterns, limitations, and possible sources of heterogeneity.

### Assessment of risk of bias

Two investigators (LAMVS and FEZM) independently examined the risk of bias in all studies that met the inclusion criteria in this systematic review. The tool proposed by Munn et al. (11), specifically designed for research reporting prevalence, was employed due to its relevance in systematic reviews and wide acceptance as a standard for methodological evaluation.

This tool covers ten key aspects of the methodology of prevalence studies, such as the representativeness of the sample about the population of interest, the suitability of the sampling frame and method, the procedure for selecting participants, minimization of non-response, direct data collection from the subjects studied, clarity of case definition, reliability and validity of measurement instruments, uniformity in the way information is collected, adequacy of the prevalence period, and appropriateness of the denominator used.

For each of these criteria, reviewers classified the risk of bias as “Low risk,” “High risk,” or “Unclear.” To quantify methodological quality globally, one point was awarded for each criterion evaluated as “Low risk.” Thus, three score ranges were established to categorize the level of bias: studies with 0–3 points were considered high risk, those with 4–6 points moderate risk, and those that reached 7–10 points were classified as low risk of bias. In case of disagreement between the two reviewers, a third researcher (JJBC) was consulted to issue a final determination, guaranteeing the evaluation process's transparency and rigor.

### Statistical analysis

The statistical software R (version 4.2.2) was used for the quantitative synthesis of results. First, the necessary data for the prevalence meta-analysis were extracted from each study: the total sample size (*n*) and the number of cases (*r*). The combination of proportions was carried out using the meta prop function of the meta package, employing the Freeman-Tukey double arcsine transformation to stabilize the variances of the proportions before their analysis.

The Clopper-Pearson method was used to calculate the 95% confidence intervals, which generate exact intervals for proportions. Due to the heterogeneity anticipated among studies—attributable to differences in population characteristics, diagnostic methods, and other contextual factors—a random-effects model was chosen following the DerSimonian and Laird approach, incorporating the Hartung-Knapp correction to adjust the confidence intervals of the effect measure.

The assessment of variability between studies was performed using the *I*² heterogeneity statistic and Cochran's *Q* test. The overall results of the meta-analysis and their respective confidence intervals were represented in forest plots. Additionally, subgroup analyses were conducted by stratifying results according to the diagnostic criteria used and by country, allowing for the examination of variability in estimates across different contexts.

Heterogeneity assessment and management was conducted using multiple approaches. Between-study heterogeneity was quantified using the *I*² statistic and assessed for statistical significance using Cochran's *Q* test. Given the anticipated substantial heterogeneity due to differences in populations, healthcare systems, diagnostic practices, and study methodologies, we employed random-effects models using the DerSimonian and Laird method with Hartung-Knapp adjustment. To explore sources of heterogeneity, we conducted pre-planned subgroup analyses stratified by diagnostic criteria and geographic regions, and performed meta-regression analyses examining the influence of publication year and sample size.

Publication bias was assessed by visual inspection of funnel plot asymmetry and formally using Egger's regression test. We acknowledge *a priori* that these methods have low statistical power and are unreliable when fewer than 10 studies are included in a meta-analysis. Therefore, formal testing for publication bias was planned only for analyses meeting this threshold.

## Results

### Selection of articles

**Figure 1:**
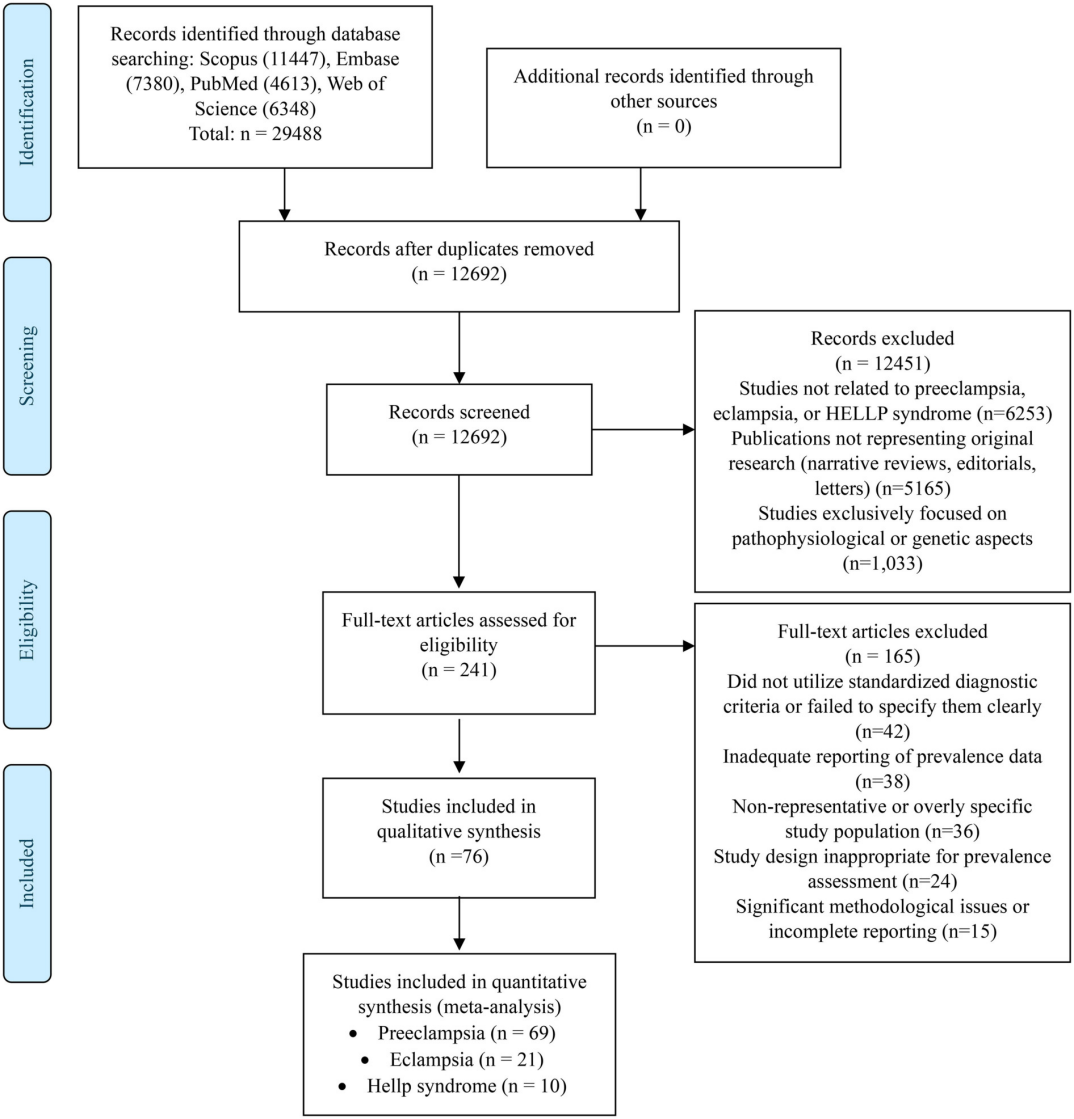
Flowchart of study selection.

The systematic search yielded 24,936 potentially relevant records. After removing duplicates, 10,692 records were screened, of which 10,451 were excluded. A full-text assessment of the remaining 241 articles led to the additional exclusion of 158 studies. Finally, 76 studies met the inclusion criteria for qualitative synthesis (14–89) (Figure 1).

### Characteristics of the studies

The final selected studies cover a broad period, ranging from the early 2000s to 2024 or 2025 (Supplementary Material 2). Collectively, they encompass diverse geographical contexts, including countries from the Americas (United States, Canada, Brazil, Argentina, Ecuador, Colombia, Uruguay, Peru, Venezuela, among others), Europe (Norway, Netherlands, Switzerland, Sweden, France, Poland, Italy, Denmark), Asia (China, Japan, South Korea, India, Pakistan, Taiwan, Malaysia, Mongolia, Iran), Africa (South Africa, Ethiopia, Algeria, Togo, Nigeria, Ghana, Tanzania), and Oceania (Australia, New Zealand).

Regarding study design, the majority employed a cross-sectional design, while a smaller group used cohort designs. A substantial number of these studies utilized hospital registry databases or national health systems, which explains the wide variability in sample size, ranging from a hundred participants (e.g., Anjum et al., with 100 pregnant women) (76) to samples exceeding one million pregnancies (e.g., Olié et al. and Lailler et al., with more than 6 and 2 million respectively) (64, 87). This allows for exploring population trends and evaluating more specific contexts, where clinical details and complementary indicators are examined in depth.

Concerning the type of sampling, a mixed distribution between probabilistic and non-probabilistic was found. Many of the studies based on official birth registries tend to employ exhaustive or representative sampling of a region or country, such as those by Wheeler et al. (75) in the United States, Tejera et al. (68) in Ecuador, or Huang et al. in Denmark (72). In contrast, some studies were conducted in referral hospitals or specific health centers, where recruitment was carried out consecutively or by convenience, such as those by Chamyan et al. (70) in Uruguay, Labarca et al. (38) in Venezuela, and Ybaseta-Medina et al. in Peru (61).

Most studies focused on singleton pregnancies, frequently excluding multiple gestations or cases with chronic hypertension and/or diabetes before 20 weeks, which aims to isolate the prevalence of preeclampsia better. However, in some broader studies, inclusion restrictions were minimal, virtually collecting all deliveries registered in a given period (22, 75)—finally, data regarding maternal age evidence a varied range. In many cases, the average is around the second half of the 20s (29, 52), although in others it rises to 35 years (88, 90). Some studies do not specify the mean age or present only broad inclusion intervals.

Regarding the diagnostic criteria for hypertensive disorders of pregnancy, the most frequently employed were those established by ACOG (14–16, 19, 25, 28, 29, 32–36, 38, 39, 41, 43, 45, 53, 63, 70, 76–78, 89) and ISSHP (17, 20–22, 24, 26, 30, 31, 36, 37, 42, 44, 46–50, 52–56, 58, 60, 64, 65, 67, 71, 72, 74, 79, 81–86, 90). Less often, some authors used the classification of the National High Blood Pressure Education Program (NHBPEP) (18, 69) or the International Classification of Diseases codes (ICD-9 and ICD-10) (23, 40, 68, 87), primarily employed in research based on hospital or administrative records.

Concerning bias analysis, it was found that the vast majority of studies obtained scores in the range of 7–8, thus placing them in the low risk of bias category. Only a few works reached 6 (moderate) values, and none were classified with lower scores. Likewise, it was observed that studies with probabilistic sampling tended to score higher systematically (14, 16, 18, 25–29, 31–35, 40, 47, 49, 50, 52–54, 58–60, 64, 65, 72, 74, 76–78, 81–84, 87, 88), thanks to the extra point awarded for that characteristic.

Studies employing probabilistic sampling methods (14, 16, 18, 25–29, 31–35, 40, 47, 49, 50, 52–54, 58–60, 64, 65, 72, 74, 76–78, 81–84, 87, 88) systematically achieved higher scores compared to those using non-probabilistic approaches, with probabilistic studies predominantly scoring 8 points due to enhanced population representativeness. These probabilistic studies included large national registry-based investigations from developed countries such as Denmark, France, Sweden, and Norway, as well as community-based surveys from developing nations including Ethiopia, Togo, and Ghana. Non-probabilistic studies were primarily hospital-based or clinic-based investigations, which typically scored 7 points despite their more limited generalizability.

The most common methodological strengths identified across studies included clear case definitions using standardized diagnostic criteria, appropriate data collection procedures, and adequate sample sizes for prevalence estimation. Common limitations were related to sampling frame representativeness, particularly among hospital-based studies that may overrepresent high-risk populations, and potential non-response bias in studies lacking detailed participation rate reporting. Individual study bias assessments and detailed scoring are provided in Supplementary Materials 2, 10.

### Meta-analysis and meta-regression of global preeclampsia prevalence

In the global meta-analysis (Table 1; Supplementary Material 3), which included a total of 70 studies with 2,465,570 participants, a preeclampsia prevalence of 4.43% (95% CI: 3.73%–5.20%) was obtained under a random-effects model, with an *I*² value of 100% (14, 15, 17–42, 44, 45, 47–55, 57–72, 74–84, 87–89). When examining subgroups according to diagnostic criteria, the ACOG group (26 studies, 2,106,907 participants) reached a combined prevalence of 4.6% (95% CI: 3.5%–5.9%) (14, 15, 19, 25, 28, 29, 32–36, 38, 39, 41, 45, 51, 57, 59, 61–63, 70, 76–78, 89). In the ISSHP subgroup (33 studies, 14,132,535 participants), the estimate was 3.7% (95% CI: 2.9%–4.6%) (17, 20–22, 24, 26, 30, 31, 37, 42, 44, 46–50, 52–56, 58, 60, 64–66, 66, 67, 71, 72, 74, 79–84). The test for differences between subgroups (*p* < 0.01) demonstrated significant heterogeneity when comparing prevalences according to the type of diagnostic criteria.

**Table 1:** Global prevalence of preeclampsia, eclampsia, and HELLP syndrome.

Condition	Criteria diagnosis	Number of studies	Total	Prevalence (%) (95% CI)	I2
Preeclampsia	ACOG	26	2,106,907	4.68 (3.51–6.01)	100%
	ISSHP	35	14,132,535	3.66 (2.82–4.61)	100%
	ICD-9	1	29,398	3.83 (3.75–3.84)	–
	NMBR	1	28,192	5.29 (4.99–5.51)	100%
	ICD-10	3	397,658	3.34 (1.97–5.07)	–
	NHBPEDP	2	7,463	6.52 (0.001–92.92)	100%
	USPSTF	1	3,695,019	8.12 (7.79–7.85)	
	WHO	1	138	14.94 (9.37–21.70)	
	Global	70	**24,655**,**710**	**4.43** (**3.73–5.20)**	100%
Eclampsia	ACOG	10	423,859	0.30 (0.09–0.61)	100%
	ISSHP	10	8,914,407	0.59 (0.12–1.41)	100%
	ICD-10	1	494,045	0.25 (0.23–0.26)	–
	Global	21	**9**,**832,311**	**0.43** (**0.19–0.76)**	100%
HELLP Syndrome	ACOG	1	2,446	0.16 (0.05–0.43)	96%
	ISSHP	8	6,412,285	0.42 (0.16–0.82)	–
	Global	**9**	**6**,**414,731**	**0.39** (**0.16–0.72)**	96%

ACOG, American college of obstetricians and gynecologists; ISSHP, international society for the study of hypertension in pregnancy; NHBPEP, national high blood pressure education program; ICD-9, international classification of diseases, 9th revision; USPSTF, United States preventive services task force; ICD-10, international classification of diseases, 10th Revision; NMBR, national maternal birth registry; WHO, World Health Organization.

Bold values indicate the global pooled results (total population and overall prevalence) for each condition.

**Figure 2:**
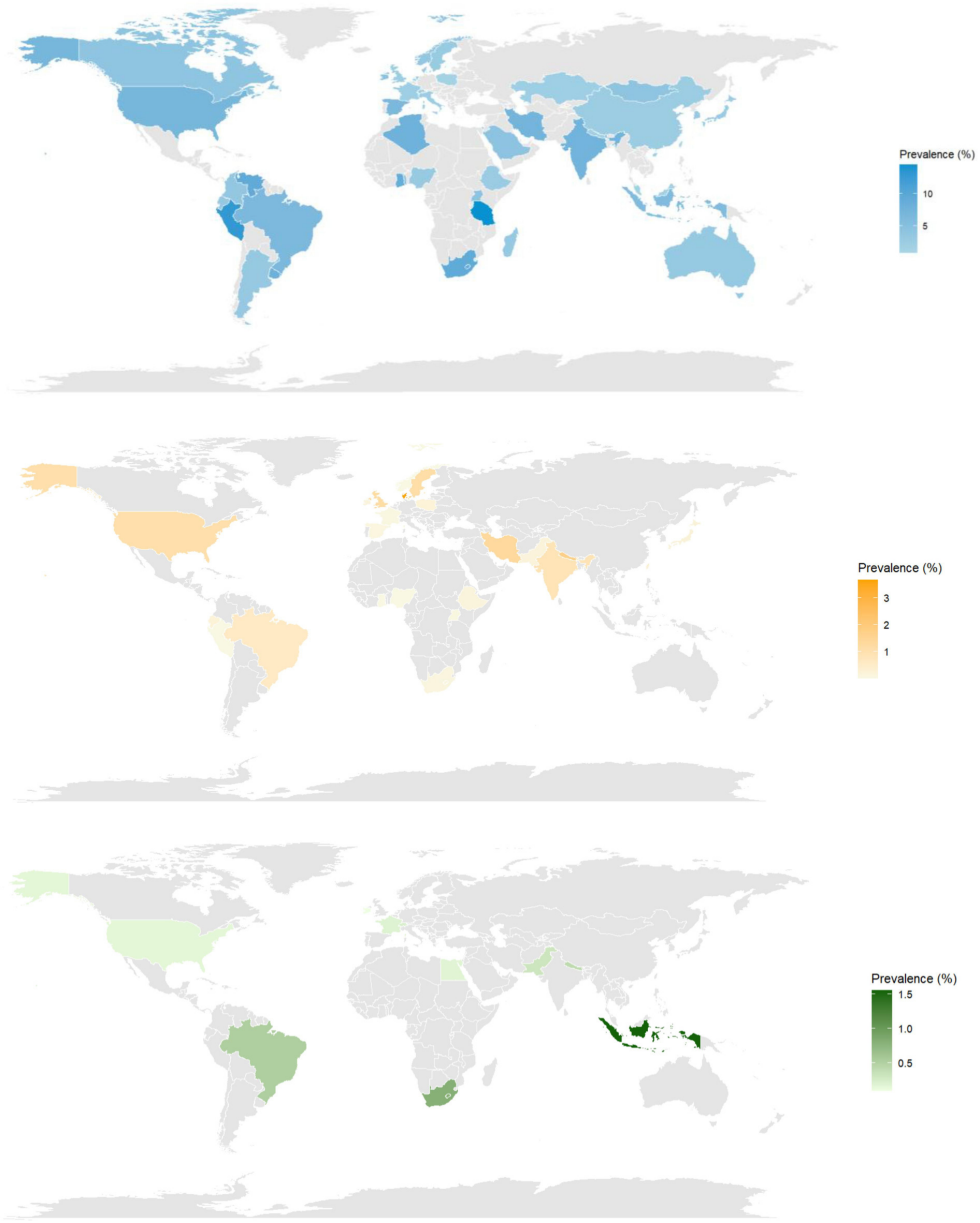
World map of the prevalence of preeclampsia (top), eclampsia (middle) and hellp syndrome (bottom). World maps showing global distribution of hypertensive disorders of pregnancy by country. Green shading intensity represents prevalence percentage as indicated in the legend. Grey areas indicate countries with no available data from included studies.

When the prevalence of preeclampsia is represented on a world map (Figure 2), the geographical variation of the disease among countries included in the review becomes evident. The map shows higher prevalence values in countries such as Peru (61), Tanzania (88), and some located in sub-Saharan Africa (17, 26, 29, 30, 67, 81, 82, 88), where they exceed even 10%. For a detailed breakdown of estimates by country, see Supplementary Material 6.

**Figure 3:**
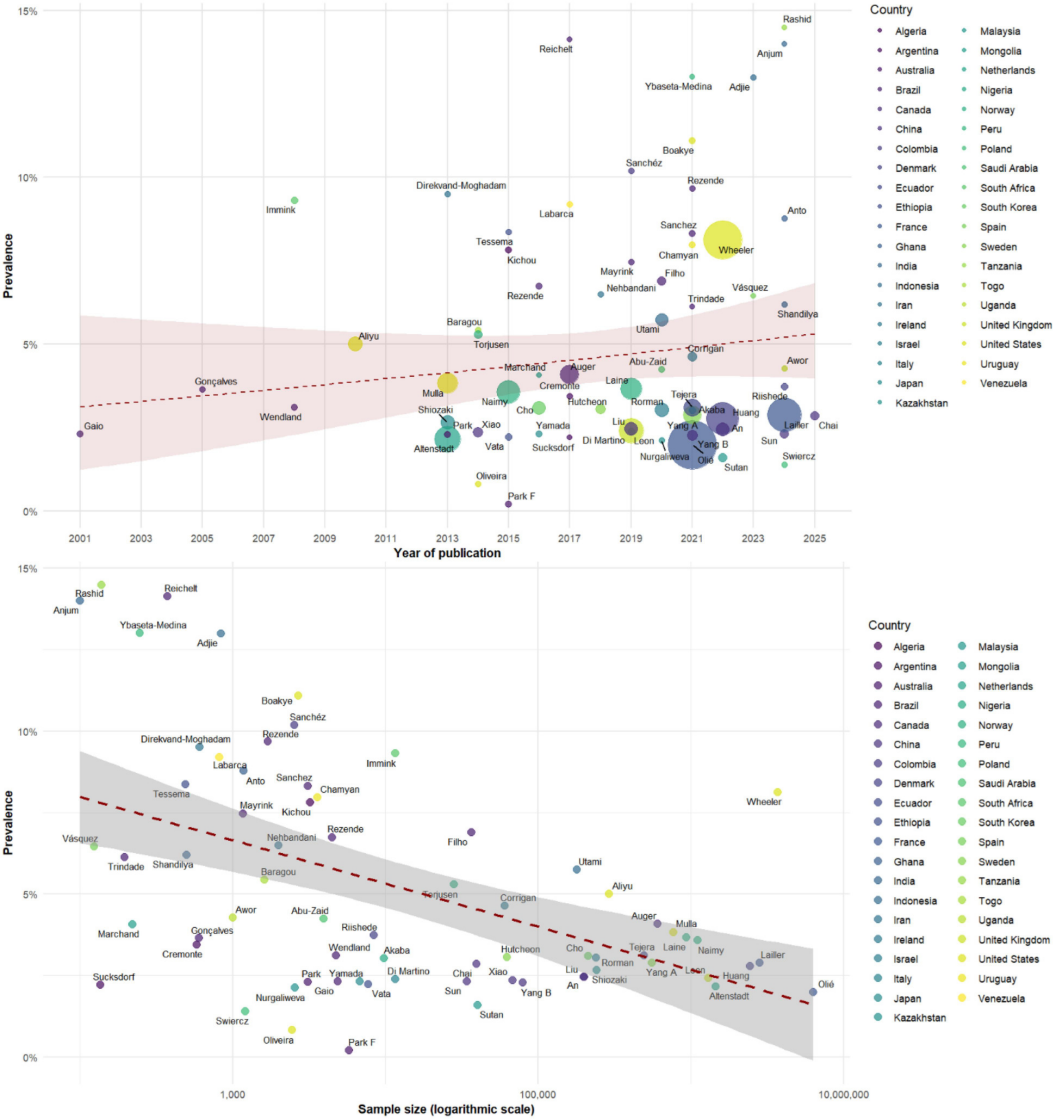
Meta-regression of the prevalence of preeclampsia by year (top) and sample size (bottom).

The meta-regression analysis examining preeclampsia prevalence across 69 studies found no statistically significant temporal trend related to publication year (coefficient = 0.0022, *p* = 0.2308). When examining the relationship between sample size and prevalence estimates, a negative association was detected that approached statistical significance (coefficient = −0.0036, *p* = 0.0524), suggesting that smaller studies tended to report higher preeclampsia prevalence rates (Figure 3).

### Meta-analysis and meta-regression of global eclampsia prevalence

This meta-analysis on eclampsia includes 21 studies with 9,832,311 participants (Table 1; Supplementary Material 4), showing a global prevalence of 0.43% (95% CI: 0.19%–0.76%) under random effects, with extremely high heterogeneity (*I*² = 100%) (14, 16, 17, 19, 38, 39, 41–43, 46, 53, 60, 64, 67–70, 72, 79, 85, 86). Results vary significantly according to diagnostic criteria, such as ACOG (0.3%) (16, 19, 38, 39, 42, 43, 53, 57, 70) and ISSHP (0.54%) (17, 46, 60, 64, 67, 72, 79, 85, 86, 91).

In the updated synthesis by country, Pakistan had the highest point estimate (2.02%); however, this result is highly uncertain, as reflected by an extremely wide confidence interval (95% CI: 0.00%–48.84%), and should be interpreted with caution (85). This was followed by high rates in Nepal (1.77%) (46, 56, 73), Egypt (1.15%) (43), and Venezuela (1.23%) (38). Other countries showed intermediate values, such as Uruguay (0.31%) (70), Ecuador (0.25%) (68), and Iran (0.60%) (42), while the combined proportion in the United States was close to 0.11% (19). Lower estimates were observed in countries like Denmark and Argentina (0.17%) (39). For a detailed breakdown of estimates by country, see Supplementary Material 6.

**Figure 4:**
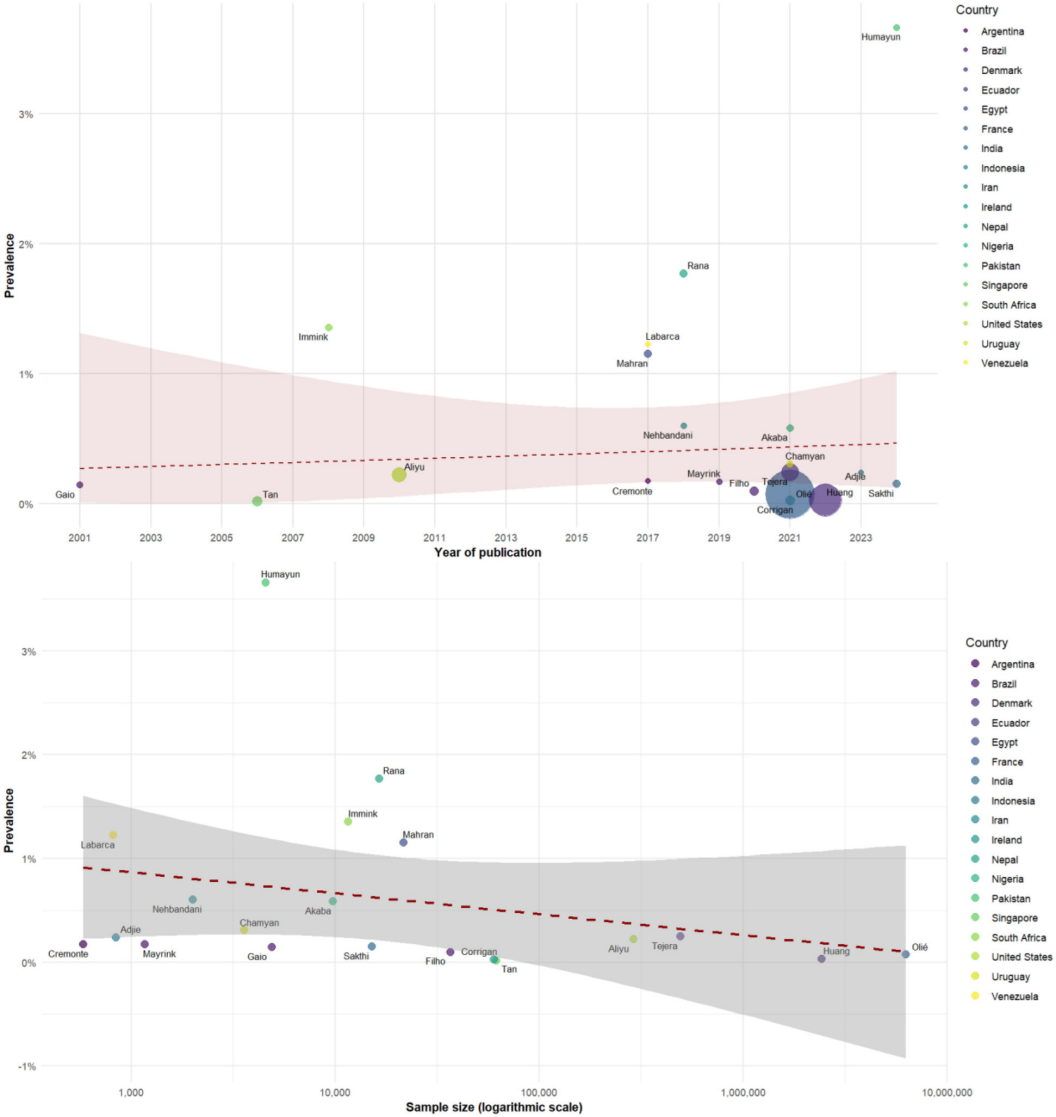
Meta-regression of the prevalence of eclampsia by year (top) and sample size (bottom).

The meta-regression analysis of 20 studies on eclampsia prevalence found no statistically significant association with publication year (coefficient = 0.0007, *p* = 0.6871). Similarly, no significant association was detected between sample size and prevalence estimates (coefficient = −0.0023, *p* = 0.6583) (Figure 4).

### Meta-analysis and meta-regression of global HELLP syndrome prevalence

In the global meta-analysis of HELLP syndrome (Table 1; Supplementary Material 5), which encompassed nine studies and a total of 6,414,731 participants, a combined prevalence of 0.37% (95% CI: 0.15%–0.69%) was obtained under a random-effects model (*I*² = 96%, *p* < 0.01) (17, 28, 53, 56, 60, 64, 73, 79, 85). When stratified by diagnostic criteria, the ISSHP subgroup included nine studies (6,412,285 participants) and reached a prevalence of 0.42% (95% CI: 0.16%–0.82%) (17, 28, 53, 56, 60, 73, 79, 85), while the ACOG subgroup, represented by a single study (2,446 participants), recorded 0.16% (95% CI: 0.05%–0.43%) (28). The test for differences between subgroups (*p* = 0.17) did not show significant variations in the prevalence estimate according to the criteria used.

Data on HELLP syndrome were more limited, as only a small number of countries had available estimates. Among them, Indonesia presented the highest proportion, with 1.55% (95% CI: 0.81–2.51) (79), followed closely by South Africa (0.80%; 95% CI 0.65–0.97) (17) and Brazil (0.52%; 0.17–1.02) (14, 53, 57). For more detail, refer to the table on eclampsia in Supplementary Material 6.

**Figure 5:**
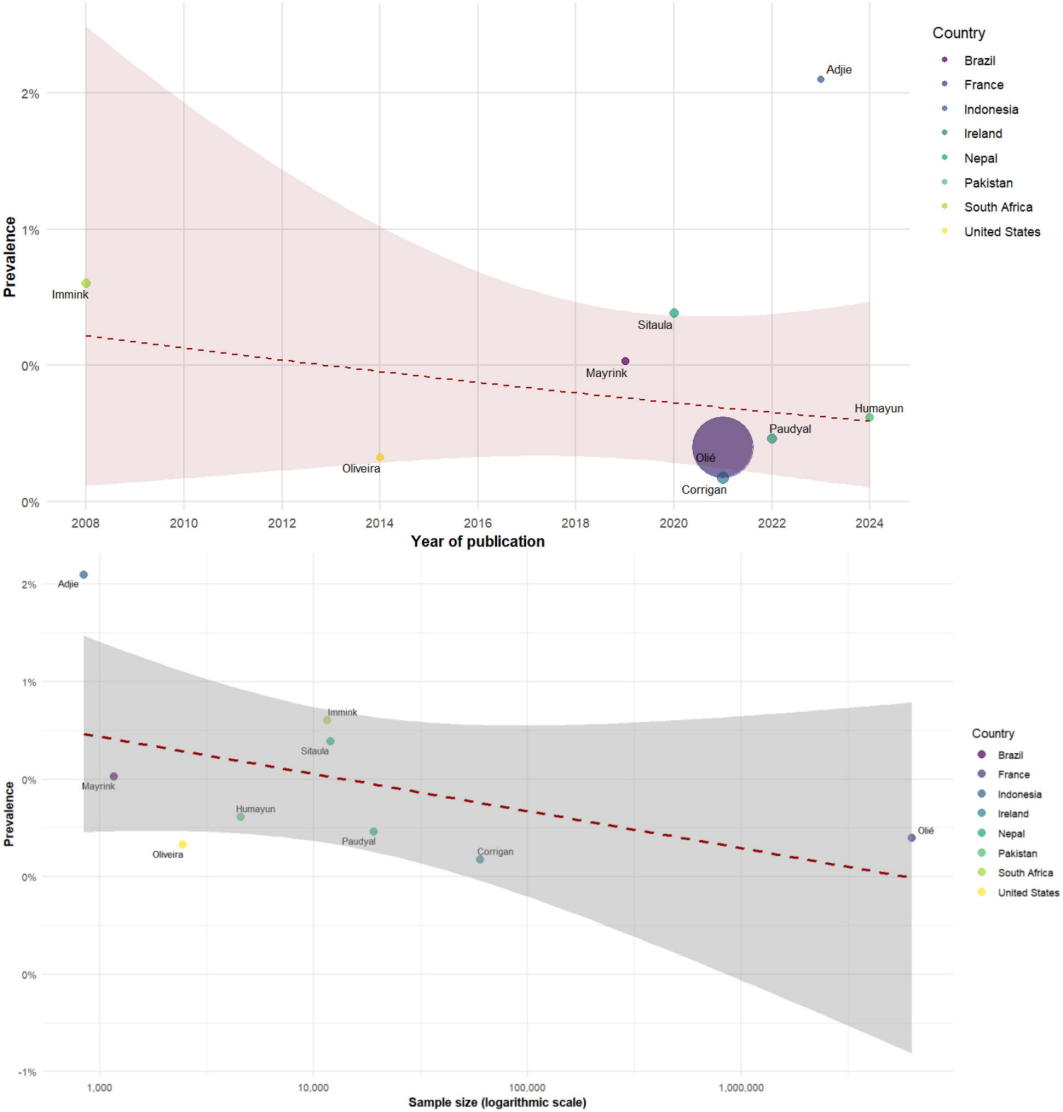
Meta-regression of Hellp syndrome by year (top) and sample size (bottom).

For HELLP syndrome, the meta-regression analysis did not detect a significant temporal trend across publication years (coefficient = −0.0015, *p* = 0.4588). The model explained 29.90% of the heterogeneity between studies (*R*² = 29.90%), with substantial residual heterogeneity remaining (*I*² = 95.67%, *p* < 0.0001). When examining the relationship between sample size and prevalence estimates, no significant association was detected (coefficient = −0.0006, *p* = 0.9437) (Figure 5).

### Regional patterns and geographic disparities

Regional synthesis across all hypertensive disorders revealed consistent patterns of geographic disparities. For preeclampsia, sub-Saharan African countries demonstrated the highest prevalences, with Tanzania reporting 14.49% (95% CI: 9.08%–21.49%) and South Africa 9.31% (95% CI: 8.79%–9.86%), while Latin American countries such as Peru 13.01% (95% CI: 9.07%–17.86%) and Venezuela 9.19% (95% CI: 7.30%–11.38%) also showed elevated rates. Similarly, eclampsia prevalences were highest in South Asian and sub-Saharan African regions, with Pakistan leading at 2.02% (95% CI: 0.00%–48.84%), followed by Nepal 1.77% (95% CI: 1.57%–1.98%) and South Africa 1.36% (95% CI: 1.15%–1.57%). For HELLP syndrome, Indonesia presented the highest prevalence at 1.55% (95% CI: 0.81%–2.51%), followed by South Africa 0.80% (95% CI: 0.65%–0.97%) and Brazil 0.52% (95% CI: 0.17%–1.02%). In contrast, European and other high-income countries consistently reported lower prevalences across all three conditions, exemplified by Denmark's eclampsia rate of 0.03% (95% CI: 0.03%–0.03%), Poland's preeclampsia rate of 1.40% (95% CI: 0.82%–2.24%), and France's HELLP syndrome rate of 0.20% (95% CI: 0.20%–0.20%). These consistent patterns reflect underlying differences in healthcare infrastructure, access to quality prenatal care, and socioeconomic determinants of maternal health across global regions (detailed country-specific data in Supplementary Material 6).

### Assessment of publication bias

Funnel plot analysis was conducted to evaluate potential publication bias across the three hypertensive disorders (Supplementary Materials 7–9). For preeclampsia studies (*n* = 70), the funnel plot showed a relatively symmetric distribution around the pooled estimate, though some asymmetry was observed with a slight paucity of smaller studies with lower prevalence rates on the left side of the plot. Egger's regression test indicated potential publication bias (*p* = 0.03), suggesting that smaller studies with higher prevalence estimates may be overrepresented in the literature. For eclampsia studies (*n* = 21), the funnel plot demonstrated moderate asymmetry with several studies falling outside the expected distribution, and Egger's test confirmed significant publication bias (*p* = 0.02). The HELLP syndrome analysis (*n* = 9) showed limited interpretability due to the small number of included studies, precluding formal statistical testing for publication bias. Overall, these findings suggest that publication bias may partially contribute to the observed prevalence estimates, particularly for preeclampsia and eclampsia, and should be considered when interpreting the pooled results.

## Discussion

### Main findings

This study represents a comprehensive and updated global meta-analysis on the prevalence of hypertensive disorders of pregnancy. At its core, the high prevalence of these conditions reflects a widespread burden of underlying endothelial dysfunction and thromboinflammatory pathways, which are central to their pathogenesis. While building upon foundational prior reviews, our work provides a unique contribution by synthesizing a larger body of more recent evidence and, for the first time, systematically analyzing the impact of differing diagnostic criteria (e.g., ACOG vs. ISSHP) on prevalence estimates. Our findings reveal consistent epidemiological patterns but with notable heterogeneity among regions. The variability observed between ACOG and ISSHP diagnostic criteria, a key focus of our analysis, suggests a significant impact of diagnostic methodology on reported estimates. The higher prevalence in low and middle-income countries reflects important disparities in social determinants of maternal health. At the same time, the inverse correlation between study sample size and reported prevalence highlights methodological biases that should be considered when interpreting the literature. These patterns confirm the multifactorial complexity of these disorders and underscore the need to standardize research methodologies to obtain more precise estimates that can adequately guide health policies.

### Geographical variations and determining characteristics

Our meta-analysis reveals marked regional differences in the prevalence of preeclampsia, with significantly higher rates in African and Latin American countries compared to those observed in Europe and North America. This geographical pattern is consistent with the findings of Abalos et al. (92), who reported that the incidence of preeclampsia was higher in low- and middle-income countries. In the particular case of eclampsia, our results also show a concentration of high prevalences in countries such as Pakistan, Nepal, and some states in sub-Saharan Africa, reflecting similar regional disparities to those described by Vousden et al. (93) in their multicenter study

Beyond these geographical patterns, it is important to systematically compare our pooled estimates with those from prior syntheses. Our higher pooled global prevalence of 4.43% for preeclampsia, when compared to earlier estimates, is likely attributable to several factors: our inclusion of a large number of studies published in the last decade, a period during which risk factors such as maternal age and obesity have increased globally, and the broader application of more sensitive diagnostic criteria (e.g., ACOG), a factor our study uniquely stratifies and analyzes. Similarly, our global eclampsia prevalence of 0.43% reflects a wider geographical scope than previous regional studies, incorporating data from diverse settings that contribute to a different summary measure. These comparisons underscore that our work builds upon previous estimates by providing an updated and methodologically stratified picture of the global burden.

The marked regional disparities identified in our analysis, such as the particularly high prevalence rates in several sub-Saharan African and Latin American countries, can be largely attributed to an interplay of socioeconomic, healthcare, and clinical factors. The elevated prevalence we found in nations like Tanzania (14.49%) and Peru (13.01%) likely reflects not only challenges in access to quality prenatal care and timely diagnosis (94, 95) but also a higher underlying burden of risk factors. These include nutritional deficiencies and a growing prevalence of comorbidities such as obesity and pre-existing hypertension, which elevate the baseline risk in these populations (95, 96). Therefore, the variations in our estimates are not just numbers; they are a reflection of deep-seated differences in public health infrastructure and population health profiles.

Epidemiological surveillance and registration system variability could also contribute to the observed differences. In countries with robust health registration systems, such as Denmark and Sweden, where our analysis found relatively low prevalences (0.03% and 0.05%, respectively, for eclampsia), diagnostic precision and registration comprehensiveness could generate more reliable estimates. In contrast, the extremely high prevalences reported in some studies from countries like Venezuela and Tanzania could reflect not only a truly higher disease burden but also a selection bias toward tertiary referral centers, as suggested by Khedagi and Bello in their critical analysis of epidemiological studies on hypertensive disorders of pregnancy (97).

These methodological considerations highlight critical limitations in directly comparing pooled prevalences across regions with fundamentally different healthcare contexts. The substantial variations we observe may reflect not only true epidemiological differences but also systematic differences in case ascertainment methods, diagnostic capacity, and reporting quality between healthcare systems. While our regional synthesis provides valuable insights for public health planning, readers must interpret these comparisons with awareness that apparent disparities may partially result from methodological heterogeneity rather than solely representing genuine differences in disease burden across populations.

The prevalence estimates identified in our analysis do not exist in a vacuum; they are intrinsically linked to global trends in maternal risk factors. The documented rise in conditions such as advanced maternal age, obesity, and pre-existing comorbidities like chronic hypertension directly contributes to the high baseline risk for preeclampsia in many populations. Therefore, our finding of a 4.43% global prevalence is not merely a static figure but reflects a dynamic public health challenge influenced by these evolving demographics. Furthermore, the significance of this prevalence extends far beyond acute pregnancy complications. A diagnosis of preeclampsia serves as a sentinel event, unmasking a woman's predisposition to future cardiovascular disease and placing her and her offspring at a higher lifetime risk for metabolic and hypertensive disorders. Thus, accurate prevalence data is critical not only for obstetric planning but also for informing long-term primary prevention strategies for chronic diseases in a substantial portion of the female population.

A critical limitation of our analysis is the underrepresentation of data from low-resource countries, particularly those with the highest burden of maternal mortality where hypertensive disorders likely contribute most significantly to adverse outcomes. Our systematic search yielded limited studies from sub-Saharan Africa, rural Asia, and other resource-constrained settings, creating a geographical bias that may underestimate the true global prevalence of these conditions. This data scarcity likely reflects multiple barriers including limited research infrastructure, inadequate funding for epidemiological studies, challenges in standardized data collection systems, and reduced opportunities for international publication from these regions. The resulting publication bias means that our estimates may not adequately represent populations at highest risk, where factors such as nutritional deficiencies, limited prenatal care access, and delayed diagnosis could result in higher true prevalences than captured in our analysis. Furthermore, under-ascertainment in low-resource settings due to insufficient diagnostic capacity, incomplete birth registries, and healthcare access barriers suggests that even available studies from these regions may underestimate disease burden. However, this limitation paradoxically represents an important contribution to the global research landscape by systematically documenting the extent of data gaps in regions where hypertensive disorders likely pose the greatest threat to maternal health. Our findings serve as a critical call to action for countries lacking robust epidemiological data to prioritize research initiatives addressing these conditions. While extrapolation of our estimates to unrepresented populations would be inappropriate, our analysis provides a foundational reference point that can guide resource allocation for targeted studies in data-scarce regions. The identification of these evidence gaps challenges the assumption that preeclampsia research is comprehensively global and highlights the urgent need for standardized surveillance systems in low-resource settings, potentially catalyzing international collaboration and funding initiatives to address these critical knowledge deficits.

Finally, it is crucial to emphasize that although the considerable heterogeneity in our data might invite skepticism, we contend that this very diversity is a fundamental strength, not a limitation, of our analysis. We acknowledge that combining studies with different diagnostic approaches generates inherent heterogeneity, as these populations represent distinct clinical entities. However, this approach reflects the current reality of research in this field: there is simply insufficient literature using standardized criteria to conduct meaningful analyses within homogeneous diagnostic categories. Our decision to provide pooled estimates serves a practical public health purpose. Policymakers often require approximate prevalence figures for resource allocation and intervention planning, even when ideal methodological conditions are not met. We believe that our comprehensive sensitivity analyses (Table 1) strengthen, rather than weaken, our contribution by allowing readers to examine estimates across different diagnostic approaches. This methodological transparency enables evidence-based decision-making while acknowledging the limitations inherent in the current state of the literature. Rather than withholding potentially useful information due to methodological puritanism, we have chosen to present these estimates with appropriate caveats, thereby providing stakeholders with the best available evidence for informed healthcare planning and policy development.

### Temporal trends, sample size, and diagnostic criteria

Our temporal meta-regression did not detect a statistically significant increase in the reported prevalence of preeclampsia and eclampsia over the last two decades. This finding should be interpreted cautiously, as it contrasts with other large population studies, such as that by Ananth et al. (98), which have documented a significant rise in prevalence over a similar period. The apparent lack of a significant trend in our analysis could reflect real epidemiological stability in the included studies or evolutions in diagnostic and registration practices that our model could not fully capture. As noted by Mol et al. (99), the progressive implementation of more sensitive diagnostic criteria and the increased use of biomarkers have broadened the spectrum of detected cases, especially in non-severe forms of preeclampsia.

Regarding the effect of sample size, our meta-regression identified a statistically significant inverse correlation between sample size and the reported prevalence of the three hypertensive disorders analyzed. Studies with smaller samples reported considerably higher rates, which several factors could explain. First, as suggested by Zwart et al. (100), smaller studies are often conducted in tertiary or referral centers with a higher concentration of complex cases, introducing selection bias. Second, according to the analysis by Thangaratinam et al. (101), smaller studies with negative results or low prevalences are less likely to be published, creating publication bias.

The changes observed over time likely represent a combination of factors. On the one hand, there are arguments for a true increase in the incidence of hypertensive disorders of pregnancy, linked to the rise in maternal age, obesity, and comorbidities such as diabetes and chronic hypertension, as demonstrated by the longitudinal data of Roberts et al. and Valensise et al. (102, 103). On the other hand, the progressive standardization of diagnostic criteria and increased awareness of these pathologies have improved their detection and registration. Particularly illustrative is the paradigm shift following the update of the ACOG guidelines in 2013 and 2019, which eliminated proteinuria as a mandatory requirement for the diagnosis of preeclampsia, amplifying the spectrum of detected cases (8).

Regarding diagnostic criteria, our analysis shows substantial differences in estimated prevalences according to the standard employed. Studies based on ACOG criteria reported significantly higher preeclampsia prevalences (4.7%) than those based on ISSHP (3.5%). These discrepancies reflect the conceptual differences between both systems. While ACOG emphasizes target organ involvement as an alternative to proteinuria, ISSHP maintains stricter thresholds for certain biochemical parameters. As proposed by Magee et al., although both systems have validity and scientific support, ISSHP offers greater specificity while ACOG privileges sensitivity (9). For clinical contexts, ACOG's more inclusive approach might favor early detection and prevention of complications, while for epidemiological research, ISSHP criteria might offer greater consistency between studies. Finally, the use of coding systems such as ICD-9 or ICD-10 in studies based on administrative records showed the lowest prevalences, probably due to undercoding, as documented by Lain et al. in their validation of diagnostic codes for hypertensive disorders (104).

Finally, the inclusion of studies with differing sampling designs presents important interpretive limitations that must be acknowledged. Our analysis combined hospital-based studies, which typically recruit from tertiary referral centers with higher concentrations of high-risk pregnancies, with population-based investigations that capture the full spectrum of obstetric care across healthcare systems. This methodological heterogeneity introduces systematic bias in prevalence estimates, as hospital-based studies inherently overrepresent severe cases and complications compared to community-based or registry studies that reflect true population prevalence. The restricted application of combining these diverse sampling approaches means that our pooled estimates may not accurately represent either hospital-based prevalence or true population prevalence, but rather a hybrid estimate influenced by the proportion of each study type included. While this limitation complicates direct clinical application of our findings, it reflects the current reality of epidemiological research in this field, where standardized population-based surveillance remains limited in many regions. Future research should prioritize population-based designs with standardized case ascertainment to provide more precise prevalence estimates for public health planning and clinical guideline development.

### Implications for public health and clinical practice

Our results reinforce the need for standardized protocols for prenatal care that emphasize early detection of risk factors and preclinical signs of preeclampsia, aligning with recent state-of-the-art clinical guidelines on screening and management (105). In line with the recommendations of Magee et al. (106), monitoring should intensify from the 20th week of gestation, with regular evaluations of blood pressure, proteinuria, and relevant biochemical parameters. Furthermore, the implementation of validated predictive tools, such as those synthesized in recent meta-analyses on outcome prediction (107), could allow for stratification of individual risk and personalization of follow-up, building upon foundational models like that of Poon et al. (108).

Our study suggests the need for adapted and feasible interventions for regions with high prevalence, especially in resource-limited settings. Implementing decentralized models of prenatal care, such as those evaluated by von Dadelszen et al. in their CLIP study (Community-Level Interventions for Pre-eclampsia), could improve access to essential services in rural or marginalized areas (109). Likewise, the training of community health workers in identifying warning signs and timely referral has been shown to significantly reduce serious complications, as evidenced by the multi-country study by Bellad et al. (110). Telemedicine represents another promising strategy, allowing remote monitoring of patients and specialized consultation in regions with a shortage of obstetricians, as documented by Lanssens et al. in their evaluation of remote monitoring platforms for high-risk pregnancies (111).

### Strengths and limitations

Among the main strengths of this study is its broad global scope, which included data from 76 investigations from diverse geographical contexts, thus providing the most comprehensive synthesis to date on the epidemiology of hypertensive disorders of pregnancy. Including more than 24 million pregnant women in the preeclampsia analysis confers robust statistical power to our estimates; however, we acknowledge that a few massive registry studies largely drive this figure and does not necessarily equate to global representativeness. Additionally, the analyses stratified by diagnostic criteria and the meta-regression by publication year and sample size allow a better understanding of the sources of heterogeneity, offering a more nuanced interpretation of the results. Another key strength is the systematic application of a validated risk-of-bias tool (Munn et al.) specific to prevalence studies, which provides a transparent framework for assessing methodological quality. However, the resulting high proportion of studies classified as “low risk” must be interpreted with caution. This often reflects strong performance in procedural domains while coexisting with significant limitations in sampling methods that affect external validity, as discussed previously.

Nevertheless, our study presents several important limitations. The extremely high heterogeneity (*I*² = 100%) in all analyses reflects substantial variability in methodologies, populations, and diagnostic criteria among primary studies, complicating the interpretation of pooled estimates. Meta-regression analyses were necessarily limited to publication year and sample size, as these were the only variables consistently reported across all included studies. Other potentially relevant variables such as maternal age, socioeconomic status, parity, and comorbidities were either not reported uniformly, presented in different formats (means vs. medians, different age categories), or available in only subsets of studies, precluding meaningful meta-regression analysis. Despite our efforts to include studies from all regions, a geographical imbalance persists with lower representation from low-income countries, particularly from sub-Saharan Africa and South Asia, where the burden of these disorders might be greater. Changes in diagnostic criteria over time, particularly differences between ACOG and ISSHP definitions, make it difficult to directly compare studies, even when they use the same general standard but in different versions. Additionally, many studies did not adequately report important risk factors such as maternal age, parity, or comorbidities, which prevented conducting adjusted analyses for these potential confounders. Finally, the small number of included studies for the HELLP syndrome analysis (*n* = 9) precluded a meaningful assessment of potential publication bias, meaning this limitation could not be formally evaluated for that specific outcome.

## Conclusions and recommendations

This global meta-analysis provides updated estimates on the prevalence of preeclampsia (4.43%), eclampsia (0.43%), and HELLP syndrome (0.39%). These figures must be interpreted with caution, given the substantial heterogeneity between studies, particularly for the HELLP syndrome estimate, which is derived from a sparse evidence base. The observed prevalences reflect considerable variation by diagnostic criteria and population characteristics, with marked disparities between high and low-resource regions that may partially reflect differences in data quality rather than true epidemiological variation. Our findings suggest that hypertensive disorders of pregnancy represent an important public health concern globally, with temporal trends indicating either increasing disease burden or improved detection systems. The variations according to diagnostic criteria underscore the need to standardize definitions, while the inverse correlation between sample size and reported prevalence highlights methodological factors that affect interpretation. This lack of a single, universally adopted diagnostic standard remains a primary obstacle to reliable international surveillance and cross-country comparisons.

In light of these results, we recommend a comprehensive approach addressing both immediate clinical needs and long-term research priorities. Strengthening epidemiological surveillance systems is essential, particularly in underrepresented regions such as sub-Saharan Africa and South Asia where disease burden appears highest but reliable data remain scarce, requiring investment in population-based registries with standardized diagnostic criteria. Future research should prioritize large-scale, multi-country collaborative studies using uniform methodologies to distinguish true epidemiological differences from methodological artifacts, while examining how healthcare system characteristics influence reported prevalences. The scientific community must establish consensus on standardized diagnostic definitions and reporting frameworks for epidemiological research, including minimum data elements and uniform population characteristic reporting. Finally, international funding agencies should prioritize maternal health surveillance research in data-scarce regions, supporting sustainable epidemiological infrastructure that enables comprehensive understanding necessary to effectively address the global burden of hypertensive disorders of pregnancy and guide evidence-based policies for reducing associated morbidity and mortality.

## Data Availability

The data analyzed in this study is subject to the following licenses/restrictions: data are available upon request to the corresponding author. Requests to access these datasets should be directed to victor.vera@untrm.edu.pe.

## References

[1] American College of Obstetricians and Gynecologists (ACOG). ACOG Practice bulletin No. 202: gestational hypertension and preeclampsia. Obstet Gynecol. (2019) 133(1):1. 10.1097/AOG.0000000000003018 30575675

[2] Abalos E Cuesta C Carroli G Qureshi Z Widmer M Vogel JP Pre-eclampsia, eclampsia and adverse maternal and perinatal outcomes: a secondary analysis of the world health organization multicountry survey on maternal and newborn health. BJOG Int J Obstet Gynaecol. (2014) 121(Suppl 1):14–24. 10.1111/1471-0528.12629 24641531

[3] Say L Chou D Gemmill A Tunçalp Ö Moller A-B Daniels J Global causes of maternal death: a WHO systematic analysis. Lancet Glob Health. (2014) 2(6):e323–33. 10.1016/S2214-109X(14)70227-X 25103301

[4] World Health Organization (WHO). (2011). WHO Recommendations for Prevention and Treatment of Pre-Eclampsia and Eclampsia. Available online at: https://www.who.int/publications/i/item/9789241548335 (Accessed January 20, 2025). 23741776

[5] Giannakou K Evangelou E Papatheodorou SI. Genetic and non-genetic risk factors for pre-eclampsia: umbrella review of systematic reviews and meta-analyses of observational studies. Ultrasound Obstet Gynecol. (2018) 51(6):720–30. 10.1002/uog.18959 29143991

[6] Pittara T Vyrides A Lamnisos D Giannakou K. Pre-eclampsia and long-term health outcomes for mother and infant: an umbrella review. BJOG Int J Obstet Gynaecol. (2021) 128(9):1421–30. 10.1111/1471-0528.16683 33638891

[7] Montgomery KS Hensley C Winseman A Marshall C Robles A. A systematic review of complications following pre-eclampsia. Matern Child Health J. (2024) 28(11):1876–85. 10.1007/s10995-024-03999-z 39316252

[8] Gestational hypertension and preeclampsia: aCOG practice bulletin, number 222. Obstet Gynecol. (2020) 135(6):e237–60. 10.1097/AOG.0000000000003891 32443079

[9] Magee LA Brown MA Hall DR Gupte S Hennessy A Karumanchi SA The 2021 international society for the study of hypertension in pregnancy classification, diagnosis & management recommendations for international practice. Pregnancy Hypertens. (2022) 27:148–69. 10.1016/j.preghy.2021.09.008 35066406

[10] Page MJ McKenzie JE Bossuyt PM Boutron I Hoffmann TC Mulrow CD The PRISMA 2020 statement: an updated guideline for reporting systematic reviews. Br Med J. (2021) 372:n71. 10.1136/bmj.n71 33782057 PMC8005924

[11] Munn Z Moola S Lisy K Riitano D Tufanaru C. Methodological guidance for systematic reviews of observational epidemiological studies reporting prevalence and cumulative incidence data. Int J Evid Based Healthc. (2015) 13(3):147–53. 10.1097/XEB.0000000000000054 26317388

[12] Cochrane. Cochrane Handbook for Systematic Reviews of Interventions. Available online at: https://training.cochrane.org/handbook (Accessed October 30, 2024).

[13] PAHO. Clasificación Estadística Internacional de Enfermedades y Problemas Relacionados con la Salud: Volumes 1, 2 & 3. 10th ed Washington, DC: Pan American Health Organization (2002). p. 1.

[14] Gaio DS Schmidt MI Duncan BB Nucci LB Matos MC Branchtein L. Hypertensive disorders in pregnancy: frequency and associated factors in a cohort of Brazilian women. Hypertens Pregnancy. (2001) 20(3):269–81. 10.1081/PRG-100107829 12044335

[15] Gonçalves R Fernandes RAQ Sobral DH. Prevalência da doença hipertensiva específica da gestação em hospital público de São Paulo. Rev Bras Enferm. (2005) 58:61–4. 10.1590/S0034-71672005000100011 16268285

[16] Tan KH Kwek K Yeo GSH. Epidemiology of pre-eclampsia and eclampsia at the KK women’s and children’s hospital, Singapore. Singapore Med J. (2006) 47(1):48–53. 16397721

[17] Immink A Scherjon S Wolterbeek R Steyn DW. Seasonal influence on the admittance of pre-eclampsia patients in tygerberg hospital. Acta Obstet Gynecol Scand. (2008) 87(1):36–42. 10.1080/00016340701743066 17963049

[18] Wendland EMDR Duncan BB Belizán JM Vigo A Schmidt MI. Gestational diabetes and pre-eclampsia: common antecedents? Arq Bras Endocrinol Metabol. (2008) 52(6):975–84. 10.1590/s0004-27302008000600008 18820808

[19] Aliyu MH Luke S Kristensen S Alio AP Salihu HM. Joint effect of obesity and teenage pregnancy on the risk of preeclampsia: a population-based study. J Adolesc Health. (2010) 46(1):77–82. 10.1016/j.jadohealth.2009.06.006 20123261

[20] Direkvand-Moghadam A Khosravi A Sayehmiri K. Predictive factors for preeclampsia in pregnant women: a receiver operation character approach. Arch Med Sci AMS. (2013) 9(4):684–9. 10.5114/aoms.2013.36900 24049529 PMC3776178

[21] Shiozaki A Matsuda Y Satoh S Saito S. Comparison of risk factors for gestational hypertension and preeclampsia in Japanese singleton pregnancies. J Obstet Gynaecol Res. (2013) 39(2):492–9. 10.1111/j.1447-0756.2012.01990.x 23002807

[22] Altenstadt JF Auf S von Hukkelhoven CWPM Roosmalen J van Bloemenkamp KWM. Pre-eclampsia increases the risk of postpartum haemorrhage: a nationwide cohort study in The Netherlands. PLoS One. (2013) 8(12):e81959. 10.1371/journal.pone.0081959 24367496 PMC3867333

[23] Mulla ZD Annavajjhala V Gonzalez-Sanchez JL Simon MR Nuwayhid BS. Group B streptococcal colonization and the risk of pre-eclampsia. Epidemiol Infect. (2013) 141(5):1089–98. 10.1017/S0950268812001598 22813482 PMC9151833

[24] Park FJ Leung CHY Poon LCY Williams PF Rothwell SJ Hyett JA. Clinical evaluation of a first trimester algorithm predicting the risk of hypertensive disease of pregnancy. Aust N Z J Obstet Gynaecol. (2013) 53(6):532–9. 10.1111/ajo.12126 23919594

[25] Xiao J Shen F Xue Q Chen G Zeng K Stone P Is ethnicity a risk factor for developing preeclampsia? An analysis of the prevalence of preeclampsia in China. J Hum Hypertens. (2014) 28(11):694–8. 10.1038/jhh.2013.148 24430700

[26] Baragou S Goeh-Akue E Pio M Afassinou YM Atta B. Hypertension artérielle et grossesse à lomé (afrique sub-saharienne): aspects épidémiologiques, diagnostiques et facteurs de risque. Ann Cardiol Angéiologie. (2014) 63(3):145–50. 10.1016/j.ancard.2014.05.006 24951092

[27] Torjusen H Brantsæter AL Haugen M Alexander J Bakketeig LS Lieblein G Reduced risk of pre-eclampsia with organic vegetable consumption: results from the prospective Norwegian mother and child cohort study. BMJ Open. (2014) 4(9):e006143. 10.1136/bmjopen-2014-006143 25208850 PMC4160835

[28] Oliveira N Doyle LE Atlas RO Jenkins CB Blitzer MG Baschat AA. External validity of first-trimester algorithms in the prediction of pre-eclampsia disease severity. Ultrasound Obstet Gynecol. (2014) 44(3):286–92. 10.1002/uog.13433 24912952

[29] Tessema GA Tekeste A Ayele TA. Preeclampsia and associated factors among pregnant women attending antenatal care in Dessie referral hospital, northeast Ethiopia: a hospital-based study. BMC Pregnancy Childbirth. (2015) 15:73. 10.1186/s12884-015-0502-7 25880924 PMC4392792

[30] Vata PK Chauhan NM Nallathambi A Hussein F. Assessment of prevalence of preeclampsia from dilla region of Ethiopia. BMC Res Notes. (2015) 8:816. 10.1186/s13104-015-1821-5 26704295 PMC4690301

[31] Park F Russo K Williams P Pelosi M Puddephatt R Walter M Prediction and prevention of early-onset pre-eclampsia: impact of aspirin after first-trimester screening. Ultrasound Obstet Gynecol. (2015) 46(4):419–23. 10.1002/uog.14819 25678383

[32] Naimy Z Grytten J Monkerud L Eskild A. The prevalence of pre-eclampsia in migrant relative to native Norwegian women: a population-based study. BJOG Int J Obstet Gynaecol. (2015) 122(6):859–65. 10.1111/1471-0528.12978 25040439

[33] Kichou B Henine N Kichou L Benbouabdellah M. Épidémiologie de la prééclampsie dans la région de tizi-ouzou (algérie). Ann Cardiol Angéiologie. (2015) 64(3):164–8. 10.1016/j.ancard.2015.04.004 26044306

[34] Cho GJ Park JH Shin S-A Oh M-J Seo HS. Metabolic syndrome in the non-pregnant state is associated with the development of preeclampsia. Int J Cardiol. (2016) 203:982–6. 10.1016/j.ijcard.2015.11.109 26625326

[35] Marchand NE Davaasambuu G McElrath TF Davaasambuu E Baatar T Troisi R. Prevalence of pregnancy hypertensive disorders in Mongolia. Pregnancy Hypertens. (2016) 6(4):413–7. 10.1016/j.preghy.2016.10.001 27939492 PMC5161111

[36] Rezende KBC de Bornia RG Esteves APVDS Cunha AJLA da Amim J Jr. Preeclampsia: prevalence and perinatal repercussions in a university hospital in Rio de Janeiro, Brazil. Pregnancy Hypertens. (2016) 6(4):253–5. 10.1016/j.preghy.2016.08.229 27939461

[37] Yamada T Obata-Yasuoka M Hamada H Baba Y Ohkuchi A Yasuda S Isolated gestational proteinuria preceding the diagnosis of preeclampsia—an observational study. Acta Obstet Gynecol Scand. (2016) 95(9):1048–54. 10.1111/aogs.12915 27109750

[38] Labarca L Urdaneta M Jr González IME Contreras Benítez A Baabel ZNS Fernández Correa M Prevalencia del síndrome de HELLP en gestantes críticas: maternidad “Dr. Armando Castillo Plaza”, Maracaibo, Venezuela. Rev Chil Obstet Ginecol. (2016) 81(3):194–201. 10.4067/S0717-75262016000300005

[39] Elizalde Cremonte A Fregenal Fuentes B Cataldi SM Pohlemann Tarnovski MC Pertiñez MYC. Morbilidad materna en pacientes con síndrome de hellp en el hospital Ángela iglesias llano corrientes capital. Rev Centroam Obstet Ginecol. (2017) 22(3):60–4. 10.37980/im.journal.revcog.3727

[40] Auger N Fraser WD Schnitzer M Leduc L Healy-Profitós J Paradis G. Recurrent pre-eclampsia and subsequent cardiovascular risk. Heart Br Card Soc. (2017) 103(3):235–43. 10.1136/heartjnl-2016-309671 27530133

[41] Bellizzi S Sobel HL Ali MM. Signs of eclampsia during singleton deliveries and early neonatal mortality in low- and middle-income countries from three WHO regions. Int J Gynaecol Obstet. (2017) 139(1):50–4. 10.1002/ijgo.12262 28704570

[42] Nehbandani S Koochakzai M Mirzaee F Moghimi F. Prevalence of preeclampsia and its maternal and fetal complications in women referring to amiralmomenin hospital of Zabol in 2014–2015. J Transl Med Res. (2017) 24(4):306–12.

[43] Mahran A Fares H Elkhateeb R Ibrahim M Bahaa H Sanad A Risk factors and outcome of patients with eclampsia at a tertiary hospital in Egypt. BMC Pregnancy Childbirth. (2017) 17(1):435. 10.1186/s12884-017-1619-7 29272998 PMC5741945

[44] Reichelt AJ Weinert LS Mastella LS Gnielka V Campos MA Hirakata VN Clinical characteristics of women with gestational diabetes—comparison of two cohorts enrolled 20 years apart in southern Brazil. Sao Paulo Med J. (2017) 135:376–82. 10.1590/1516-3180.2016.0332190317 28793129 PMC10015997

[45] Sucksdorf MVM Strada BN Abud AM Alessandría MC Gastaldi G Quaino FD Análisis de los factores de riesgo para el desarrollo de estados hipertensivos del embarazo. Rev Fed Argent Cardiol. (2017) 46(4):224–7. 10.63600/de5r6871

[46] Rana S Kattel P. Eclampsia at a tertiary care hospital of Nepal: a five year study. Janaki Med Coll J Med Sci. (2018) 6(2):14–21. 10.3126/jmcjms.v6i02.22056

[47] Hutcheon JA Stephansson O Cnattingius S Bodnar LM Wikström A-K Johansson K. Pregnancy weight gain before diagnosis and risk of preeclampsia: a population-based cohort study in nulliparous women. Hypertens Dallas Tex 1979. (2018) 72(2):433–41. 10.1161/HYPERTENSIONAHA.118.10999 PMC6043369 29915016

[48] Di Martino D Masturzo B Paracchini S Bracco B Cavoretto P Prefumo F Comparison of two “a priori” risk assessment algorithms for preeclampsia in Italy: a prospective multicenter study. Arch Gynecol Obstet. (2019) 299(6):1587–96. 10.1007/s00404-019-05146-4 30953193

[49] Leon LJ McCarthy FP Direk K Gonzalez-Izquierdo A Prieto-Merino D Casas JP Preeclampsia and cardiovascular disease in a large UK pregnancy cohort of linked electronic health records: a CALIBER study. Circulation. (2019) 140(13):1050–60. 10.1161/CIRCULATIONAHA.118.038080 31545680

[50] Laine K Murzakanova G Sole KB Pay AD Heradstveit S Räisänen S. Prevalence and risk of pre-eclampsia and gestational hypertension in twin pregnancies: a population-based register study. BMJ Open. (2019) 9(7):e029908. 10.1136/bmjopen-2019-029908 31278106 PMC6615795

[51] Liseth Salamanca Sánchez A Nieves Díaz LA Arenas Cárdenas YM. Preeclamsia: prevalencia y factores asociados en gestantes de una institución de salud de boyacá en el periodo 2015 a 2017. Rev Investig En Salud Fac Cienc Salud Univ Boyacá. (2019) 6(2):40–52. 10.24267/23897325.422

[52] Liu L Wang L Yang W Ni W Jin L Liu J Gestational hypertension and pre-eclampsia and risk of spontaneous premature rupture of membranes: a population-based cohort study. Int J Gynaecol Obstet. (2019) 147(2):195–201. 10.1002/ijgo.12943 31420867

[53] Mayrink J Souza RT Feitosa FE Rocha Filho EA Leite DF Vettorazzi J Mean arterial blood pressure: potential predictive tool for preeclampsia in a cohort of healthy nulliparous pregnant women. BMC Pregnancy Childbirth. (2019) 19(1):460. 10.1186/s12884-019-2580-4 31795971 PMC6892235

[54] Nurgaliyeva GT Semenova YM Tanysheva GA Akylzhanova ZE Bologan I Manabayeva GK. Epidemiology of pre-eclampsia in the republic of Kazakhstan: maternal and neonatal outcomes. Pregnancy Hypertens. (2020) 20:1–6. 10.1016/j.preghy.2020.02.003 32092664

[55] Utami SM Handayani F Hidayah M. Ecological analysis of preeclampsia/eclampsia case in Sidoarjo regency, Indonesia, 2015–2019. Indian J Forensic Med Toxicol. (2020) 14(4):3474–9. 10.37506/ijfmt.v14i4.12164

[56] Sitaula S Manandhar T Thapa BD Shrestha R Dharel D. Prevalence of hemolysis, elevated liver enzymes, low platelet count syndrome in pregnant women in a tertiary care hospital. JNMA J Nepal Med Assoc. (2020) 58(226):405–8. 10.31729/jnma.4921 32788757 PMC7580356

[57] Ramos Filho FL Antunes CMF de. Hypertensive disorders: prevalence, perinatal outcomes and cesarean section rates in pregnant women hospitalized for delivery. Rev Bras Ginecol E Obstet Rev Fed Bras Soc Ginecol E Obstet. (2020) 42(11):690–6. 10.1055/s-0040-1714134 PMC10309246 33254262

[58] Abu-Zaid A Alomari M Al-Hayani M Bazi A Almazmomy A Alsaegh A Advanced maternal age and the frequency of pre-eclampsia—a single-center cross sectional study from Saudi Arabia. J Evol Med Dent Sci. (2020) 9(37):2726–9. 10.14260/jemds/2020/592

[59] Rorman E Freud A Wainstock T Sheiner E. Maternal preeclampsia and long-term infectious morbidity in the offspring—a population based cohort analysis. Pregnancy Hypertens. (2020) 21:30–4. 10.1016/j.preghy.2020.04.010 32371355

[60] Corrigan L O’Farrell A Moran P Daly D. Hypertension in pregnancy: prevalence, risk factors and outcomes for women birthing in Ireland. Pregnancy Hypertens. (2021) 24:1–6. 10.1016/j.preghy.2021.02.005 33618054

[61] Ybaseta-Medina J Ybaseta-Soto M Oscco-Torres O Medina-Saravia C. Factores de riesgo para pre-eclampsia en un hospital general de ica, perú. Rev Médica Panacea. (2021) 10(1):6–10. 10.35563/rmp.v10i1.397

[62] Trindade CR Torloni MR Mattar R Sun SY. Good performance of bioimpedance in early pregnancy to predict preeclampsia. Pregnancy Hypertens. (2021) 26:24–30. 10.1016/j.preghy.2021.08.115 34469830

[63] Sanchez MP Guida JP Simões M Marangoni-Junior M Cralcev C Santos JC Can pre-eclampsia explain higher cesarean rates in the different groups of Robson’s classification? Int J Gynaecol Obstet. (2021) 152(3):339–44. 10.1002/ijgo.13370 32920856

[64] Olié V Moutengou E Grave C Deneux-Tharaux C Regnault N Kretz S Prevalence of hypertensive disorders during pregnancy in France (2010–2018): the nationwide CONCEPTION study. J Clin Hypertens Greenwich Conn. (2021) 23(7):1344–53. 10.1111/jch.14254 PMC8678732 34042277

[65] Yang Y Le Ray I Zhu J Zhang J Hua J Reilly M. Preeclampsia prevalence, risk factors, and pregnancy outcomes in Sweden and China. JAMA Netw Open. (2021) 4(5):e218401. 10.1001/jamanetworkopen.2021.8401 33970258 PMC8111481

[66] Rezende KBC de Bornia RG Rolnik DL Amim J Pritsivelis C Cardoso MIMP External validation of first trimester combined screening for pre-eclampsia in Brazil: an observational study. Pregnancy Hypertens. (2021) 26:110–5. 10.1016/j.preghy.2021.10.005 34739940

[67] Akaba GO Anyang UI Ekele BA. Prevalence and materno-fetal outcomes of preeclampsia/eclampsia amongst pregnant women at a teaching hospital in north-central Nigeria: a retrospective cross-sectional study. Clin Hypertens. (2021) 27(1):20. 10.1186/s40885-021-00178-y 34649619 PMC8518182

[68] Tejera E Sánchez ME Henríquez-Trujillo AR Pérez-Castillo Y Coral-Almeida M. A population-based study of preeclampsia and eclampsia in Ecuador: ethnic, geographical and altitudes differences. BMC Pregnancy Childbirth. (2021) 21(1):116. 10.1186/s12884-021-03602-1 33563238 PMC7874663

[69] Boakye E Sharma G Ogunwole SM Zakaria S Vaught AJ Kwapong YA Relationship of preeclampsia with maternal place of birth and duration of residence among non-hispanic black women in the United States. Circ Cardiovasc Qual Outcomes. (2021) 14(2):e007546. 10.1161/CIRCOUTCOMES.120.007546 33563008 PMC7887058

[70] Chamyan JM Chamyan M Kryzanowski V Gandulia S Salgado V Feldman F Prevalencia de preeclampsia y sus complicaciones en el hospital de clínicas: estudio observacional 2014–2018. Anales De La Facultad De Medicina. (2021) 8(s3):1–7.

[71] Sutan R Aminuddin NA Mahdy ZA. Prevalence, maternal characteristics, and birth outcomes of preeclampsia: a cross-sectional study in a single tertiary healthcare center in greater Kuala Lumpur Malaysia. Front Public Health. (2022) 10:973271. 10.3389/fpubh.2022.973271 36324467 PMC9618654

[72] Huang C Wei K Lee PMY Qin G Yu Y Li J. Maternal hypertensive disorder of pregnancy and mortality in offspring from birth to young adulthood: national population based cohort study. Br Med J. (2022) 379:e072157. 10.1136/bmj-2022-072157 36261141 PMC9580246

[73] Paudyal P Shrestha P Bajracharya S. HELLP syndrome among pregnant women delivering at a tertiary care hospital in Kathmandu: a descriptive cross-sectional study. J South Asian Fed Obstet Gynaecol. (2022) 14(2):132–5. 10.5005/jp-journals-10006-2043

[74] An H Jin M Li Z Zhang L Li H Zhang Y Impact of gestational hypertension and pre-eclampsia on preterm birth in China: a large prospective cohort study. BMJ Open. (2022) 12(9):e058068. 10.1136/bmjopen-2021-058068 36167382 PMC9516080

[75] Wheeler SM Myers SO Swamy GK Myers ER. Estimated prevalence of risk factors for preeclampsia among individuals giving birth in the US in 2019. JAMA Netw Open. (2022) 5(1):e2142343. 10.1001/jamanetworkopen.2021.42343 34982156 PMC8728614

[76] Anjum M Dongapure N Khan UR. Placental laterality as a predictor for development of preeclampsia. Indian J Public Health Res Dev. (2022) 14(1):173–7. 10.37506/ijphrd.v14i1.18825

[77] Shandilya V Sinha N Rani S. Preeclampsia: prevalence, risk factors, and impact on mother and fetus. Indian J Cardiovasc Dis Women. (2023) 8(3):193–9. 10.25259/IJCDW_32_2023

[78] Sun M Luo M Wang T Wei J Zhang S Shu J Effect of the interaction between advanced maternal age and pre-pregnancy BMI on pre-eclampsia and GDM in central China. BMJ Open Diabetes Res Care. (2023) 11(2):e003324. 10.1136/bmjdrc-2023-003324 37085280 PMC10124205

[79] Adjie JMS Angkawijaya C. Profile of preeclampsia patients with aggravating factors: a retrospective study. Open Public Health J. (2023) 16(1):e187494452308251. 10.2174/0118749445241244231123111732

[80] Vázquez S Pascual J Durán-Jordà X Hernández JL Crespo M Oliveras A. Predictors of preeclampsia in the first trimester in normotensive and chronic hypertensive pregnant women. J Clin Med. (2023) 12(2):579. 10.3390/jcm12020579 36675508 PMC9865932

[81] Anto EO Boadu WIO Ansah E Tawiah A Frimpong J Tamakloe VCKT Prevalence of preeclampsia and algorithm of adverse foeto-maternal risk factors among pregnant women in the central region of Ghana: a multicentre prospective cross-sectional study. PLoS One. (2023) 18(6):e0288079. 10.1371/journal.pone.0288079 37384786 PMC10309986

[82] Awor S Abola B Byanyima R Orach CG Kiondo P Kaye DK Prediction of pre-eclampsia at st. Mary’s hospital lacor, a low-resource setting in northern Uganda, a prospective cohort study. BMC Pregnancy Childbirth. (2023) 23(1):101. 10.1186/s12884-023-05420-z 36755228 PMC9906950

[83] Świercz G Zmelonek-Znamirowska A Armańska J Stanisławska M Mlodawska M Młodawski J. Prevalence of pregnancy complications in the Świętokrzyskie province: a follow-up of a cohort of patients participating in first-trimester prenatal screening tests. Med Stud Med. (2024) 40(4):341–6. 10.5114/ms.2024.145617

[84] Riishede I Ekelund CK Sperling L Overgaard M Knudsen CS Clausen TD Screening for pre-eclampsia with competing-risks model using placental growth factor measurement in blood samples collected before 11weeks’ gestation. Ultrasound Obstet Gynecol. (2024) 63(3):342–9. 10.1002/uog.27462 37698230

[85] Humayun J Afridi WV Shaikh AG Afzal M Hassan S Aziz U. Risk factors of eclampsia and its maternal and perinatal effects at tertiary hospital: a retrospective study. J Popul Ther Clin Pharmacol. (2024) 31:1252–9. 10.53555/jptcp.v31i4.5743

[86] Sakthi A Joycerani D Synthiah A Lakshmi MV. Prospective study of eclampsia in a tertiary care hospital. Int J Pharmaceut Clin Res. (2024) 16(2):07–13. Available online at: https://www.ijpcr.com.

[87] Lailler G Fosse-Edorh S Lebreton E Regnault N Deneux-Tharaux C Tsatsaris V Impact of different types of hypertensive disorders of pregnancy and their duration on incident post-partum risk of diabetes mellitus: results from the French nationwide study CONCEPTION. Diabetes Metab. (2024) 50(5):101564. 10.1016/j.diabet.2024.101564 39059484

[88] Rashid SA Seif SA Omar RB. Prevalence, predictors and management of pre-eclampsia among pregnant women attending antenatal clinics in Zanzibar. Tanzan J Health Res. (2024) 25(3):1132–49. 10.4314/thrb.v25i3.12

[89] Chai L Li S Yin B Zhu X Zhu B Wu K. Prevalence, risk factors, and adverse perinatal outcomes in Chinese women with preeclampsia: a large retrospective cohort study. J Health Popul Nutr. (2025) 44(1):32. 10.1186/s41043-025-00778-6 39920879 PMC11806619

[90] Vásquez-Romero LEM Zuzunaga-Montoya FE Loayza-Castro JA Vigil-Ventura E Ramos W Vera-Ponce VJ. Prevalence of obesity according to body mass index, waist circumference, and waist-to-height ratio in Peru: a systematic review and meta-analysis. Obes Pillars. (2025) 13:100166. 10.1016/j.obpill.2025.100166 40028617 PMC11869839

[91] Akhter N Iqbal M Iqbal S Khan S Safdar S Ahmad B. Seasonal incidence of eclampsia amongst pregnant women: our experience at a tertiary care hospital. Pak J Med Health Sci. (2023) 17(02):15–15. 10.53350/pjmhs202317215

[92] Abalos E Cuesta C Grosso AL Chou D Say L. Global and regional estimates of preeclampsia and eclampsia: a systematic review. Eur J Obstet Gynecol Reprod Biol. (2013) 170(1):1–7. 10.1016/j.ejogrb.2013.05.005 23746796

[93] Vousden N Lawley E Seed PT Gidiri MF Goudar S Sandall J Incidence of eclampsia and related complications across 10 low- and middle-resource geographical regions: secondary analysis of a cluster randomised controlled trial. PLoS Med. (2019) 16(3):e1002775. 10.1371/journal.pmed.1002775 30925157 PMC6440614

[94] Souza JP Gülmezoglu AM Vogel J Carroli G Lumbiganon P Qureshi Z Moving beyond essential interventions for reduction of maternal mortality (the WHO multicountry survey on maternal and newborn health): a cross-sectional study. Lancet Lond Engl. (2013) 381(9879):1747–55. 10.1016/S0140-6736(13)60686-8 23683641

[95] Hofmeyr GJ Lawrie TA Atallah ÁN Torloni MR. Calcium supplementation during pregnancy for preventing hypertensive disorders and related problems. Cochrane Database Syst Rev. (2018) 10(10):CD001059. 10.1002/14651858.CD001059.pub5 30277579 PMC6517256

[96] Brown MA Magee LA Kenny LC Karumanchi SA McCarthy FP Saito S Hypertensive disorders of pregnancy: ISSHP classification, diagnosis, and management recommendations for international practice. Hypertens Dallas Tex 1979. (2018) 72(1):24–43. 10.1161/HYPERTENSIONAHA.117.10803 29899139

[97] Khedagi AM Bello NA. Hypertensive disorders of pregnancy. Cardiol Clin. (2021) 39(1):77–90. 10.1016/j.ccl.2020.09.005 33222817 PMC7720658

[98] Ananth CV Keyes KM Wapner RJ. Pre-eclampsia rates in the United States, 1980–2010: age-period-cohort analysis. Br Med J. (2013) 347:f6564. 10.1136/bmj.f6564 24201165 PMC3898425

[99] Mol BWJ Roberts CT Thangaratinam S Magee LA de Groot CJM Hofmeyr GJ. Pre-eclampsia. Lancet Lond Engl. (2016) 387(10022):999–1011. 10.1016/S0140-6736(15)00070-7 26342729

[100] Zwart JJ Richters JM Ory F de Vries JIP Bloemenkamp KWM van Roosmalen J. Severe maternal morbidity during pregnancy, delivery and puerperium in the Netherlands: a nationwide population-based study of 371,000 pregnancies. BJOG Int J Obstet Gynaecol. (2008) 115(7):842–50. 10.1111/j.1471-0528.2008.01713.x 18485162

[101] Cnossen JS de Ruyter-Hanhijärvi H van der Post JAM Mol BWJ Khan KS ter Riet G. Accuracy of serum uric acid determination in predicting pre-eclampsia: a systematic review. Acta Obstet Gynecol Scand. (2006) 85(5):519–25. 10.1080/00016340500342037 16755708

[102] Roberts CL Ford JB Algert CS Antonsen S Chalmers J Cnattingius S Population-based trends in pregnancy hypertension and pre-eclampsia: an international comparative study. BMJ Open. (2011) 1(1):e000101. 10.1136/bmjopen-2011-000101 22021762 PMC3191437

[103] Valensise H Vasapollo B Gagliardi G Novelli GP. Early and late preeclampsia: two different maternal hemodynamic states in the latent phase of the disease. Hypertens Dallas Tex 1979. (2008) 52(5):873–80. 10.1161/HYPERTENSIONAHA.108.117358 18824660

[104] Lain SJ Hadfield RM Raynes-Greenow CH Ford JB Mealing NM Algert CS Quality of data in perinatal population health databases: a systematic review. Med Care. (2012) 50(4):e7–20. 10.1097/MLR.0b013e31821d2b1d 21617569

[105] Wu P Green M Myers JE. Hypertensive disorders of pregnancy. Br Med J. (2023) 381:e071653. 10.1136/bmj-2022-071653 37391211

[106] Magee LA Pels A Helewa M Rey E von Dadelszen P. Canadian Hypertensive disorders of pregnancy working group. Diagnosis, evaluation, and management of the hypertensive disorders of pregnancy: executive summary. J Obstet Gynaecol Can JOGC. (2014) 36(5):416–41. 10.1016/s1701-2163(15)30588-0 24927294

[107] Bucher V Mitchell AR Gudmundsson P Atkinson J Wallin N Asp J Prediction of adverse maternal and perinatal outcomes associated with pre-eclampsia and hypertensive disorders of pregnancy: a systematic review and meta-analysis. eClinicalMedicine. (2024) 76:102861. 10.1016/j.eclinm.2024.102861 39391014 PMC11465897

[108] Poon LC Shennan A Hyett JA Kapur A Hadar E Divakar H The international federation of gynecology and obstetrics (FIGO) initiative on pre-eclampsia: a pragmatic guide for first-trimester screening and prevention. Int J Gynecol Obstet. (2019) 145(S1):1–33. 10.1002/ijgo.12802 PMC6944283 31111484

[109] von Dadelszen P Bhutta ZA Sharma S Bone J Singer J Wong H The community-level interventions for pre-eclampsia (CLIP) cluster randomised trials in Mozambique, Pakistan, and India: an individual participant-level meta-analysis. Lancet Lond Engl. (. 2020) 396(10250):553–63. 10.1016/S0140-6736(20)31128-4 PMC7445426 32828187

[110] Bellad MB Vidler M Honnungar NV Mallapur A Ramadurg U Charanthimath U Maternal and newborn health in Karnataka state, India: the community level interventions for pre-eclampsia (CLIP) trial’s baseline study results. PLoS One. (2017) 12(1):e0166623. 10.1371/journal.pone.0166623 28107350 PMC5249209

[111] Lanssens D Vandenberk T Thijs IM Grieten L Gyselaers W. Effectiveness of telemonitoring in obstetrics: scoping review. J Med Internet Res. (2017) 19(9):e327. 10.2196/jmir.7266 28954715 PMC5637065

