# Supplementary material for: Global prevalence of preeclampsia, eclampsia, and HELLP syndrome: a systematic review and meta-analysis
Source: Front Reprod Health. 2025 Nov 10;7:1706009. doi: 10.3389/frph.2025.1706009 (PMC12640961; doi:10.3389/frph.2025.1706009)
Supplement: Supplementary file 1 [file Datasheet1.pdf]

Supplementary material 1. Search strategy

Search strategy in PUBMED	
#1	"Pre-Eclampsia"[Mesh] OR preeclampsia[tiab] OR "pre eclampsia"[tiab] OR "pre-eclampsia"[tiab] OR "toxemia of pregnancy"[tiab] OR "toxemia pregnancy"[tiab] OR "toxaemia of pregnancy"[tiab] OR "toxaemia pregnancy"[tiab] OR "preeclampsia"[tiab] OR "preclampsia"[tiab] OR "Eclampsia"[Mesh] OR eclampsia[tiab] OR "HELLP Syndrome"[Mesh] OR "HELLP syndrome"[tiab] OR "sindrome HELLP"[tiab] OR "síndrome HELLP"[tiab]
#2	prevalence[tiab] OR "prevalence"[Mesh] OR frequency[tiab] OR "frecuencia"[tiab] OR "prevalencia"[tiab]
#3	#1 AND #2
Search strategy in SCOPUS	
#1	TITLE-ABS-KEY (("Pre-Eclampsia" OR preeclampsia OR "pre eclampsia" OR "pre-eclampsia" OR "toxemia of pregnancy" OR "toxemia pregnancy" OR "toxaemia of pregnancy" OR "toxaemia pregnancy" OR "preeclampsia" OR "preclampsia" OR "Eclampsia" OR eclampsia OR "HELLP Syndrome" OR "HELLP syndrome" OR "sindrome HELLP" OR "síndrome HELLP")
#2	TITLE-ABS-KEY (prevalence OR epidemiology OR frequency OR frecuencia OR prevalencia)
#3	#1 AND #2
Search strategy in Web of Science	
#1	TS=("Pre-Eclampsia" OR preeclampsia OR "pre eclampsia" OR "pre-eclampsia" OR "toxemia of pregnancy" OR "toxemia pregnancy" OR "toxaemia of pregnancy" OR "toxaemia pregnancy" OR "preeclampsia" OR "preclampsia" OR "Eclampsia" OR eclampsia OR "HELLP Syndrome" OR "HELLP syndrome" OR "sindrome HELLP" OR "síndrome HELLP")
#2	TS=(prevalence OR epidemiology OR frequency OR frecuencia OR prevalencia)
#3	#1 AND #2
Search strategy inEMBASE	
#1	('preeclampsia'/exp OR 'eph gestoses':ti,ab OR 'eph gestosis':ti,ab OR 'eph syndrome':ti,ab OR 'eph toxemia':ti,ab OR 'hep syndrome':ti,ab OR 'eclamptic toxaemia':ti,ab OR 'eclamptic toxemia':ti,ab OR 'eclamptogenic toxaemia':ti,ab OR 'eclamptogenic toxemia':ti,ab OR 'edema-proteinuria-hypertension gestoses':ti,ab OR 'edema-proteinuria-hypertension gestosis':ti,ab OR 'gestational toxaemia':ti,ab OR 'gestational toxemia':ti,ab OR 'gestational toxicosis':ti,ab OR 'gestoses':ti,ab OR 'gestosis':ti,ab OR 'gestosis, eph':ti,ab OR 'maternal toxemia':ti,ab OR 'pre eclampsia':ti,ab OR 'pre-eclampsia':ti,ab OR 'pre-eclamptic':ti,ab OR 'pre-eclamptic toxaemia':ti,ab OR 'pre-eclamptic toxemia':ti,ab OR 'preclampsia':ti,ab OR 'preeclampsia':ti,ab OR 'preeclamptic':ti,ab OR 'preeclamptic toxaemia':ti,ab OR 'preeclamptic toxemia':ti,ab OR 'pregnancy toxaemia':ti,ab OR 'pregnancy toxaemias':ti,ab OR 'pregnancy toxemia':ti,ab OR 'pregnancy toxemias':ti,ab OR 'pregnancy toxicosis':ti,ab OR 'proteinuric hypertension of pregnancy':ti,ab OR 'toxaemia gravidum':ti,ab OR 'toxaemia, preeclamptic':ti,ab OR 'toxemia during pregnancy':ti,ab OR 'toxemia gravidum':ti,ab OR 'toxemia in pregnancy':ti,ab OR 'toxemia, preeclamptic':ti,ab OR 'toxemic pregnancy':ti,ab OR 'toxicosis gravidarum':ti,ab)
#2	(prevalence:ti,ab,kw OR epidemiology:ti,ab,kw OR frequency:ti,ab,kw OR frecuencia:ti,ab,kw OR prevalencia:ti,ab,kw)
#3	#1 AND #2

Supplementary Material 2. Characteristics of the selected articles on the prevalence of preeclampsia, eclampsia and HELLP syndrome

First Author	Year	Country	Study Type	Sampling	Selection Criteria	Sample	Age (Mean in years)	Risk of Bias Score
Gaio	2001	Brazil	Prospective cohort	Probabilistic	Pregnant women ≥20 years (1991-1995), without previous diabetes, multiple Brazilian cities.	4,892	27.4	8 (low)
Gonçalves	2005	Brazil	Retrospective cohort	Non-probabilistic	Women hospitalized with gestational hypertension in public hospital São Paulo (January-July 2002).	604		7 (low)
Tan	2006	Singapore	Cohort	Probabilistic	Deliveries at KK Women's and Children's Hospital (1999-2003)	61,595	—	8 (low)
Immink	2008	South Africa	Cross-sectional	Non-probabilistic	Preeclampsia/eclampsia/HELLP at Tygerberg Hospital (2002-2003)	11,585	—	8 (low)
Wendland	2008	Brazil	Prospective cohort	Probabilistic	Pregnant women ≥20 years, without previous type 2 diabetes (1991-1994), six Brazilian cities.	4,766	27.8	8 (low)
Aliyu	2010	United States	Cohort	Non-probabilistic	Women 13-24 years with BMI data and diagnosis; excluded: incomplete information	290,807	—	7 (low)
Direkvand-Moghadam	2013	Iran	Cross-sectional	Non-probabilistic	Pregnant women receiving care; excluded: abortion <20 weeks	610	—	7 (low)
Shiozaki	2013	Japan	Cohort	Non-probabilistic	Singleton pregnancies without essential hypertension, deliveries >22 weeks	241,292	—	8 (low)
Altenstadt	2013	Netherlands	Cohort	Non-probabilistic	Deliveries >19 weeks; excluded: missing data	1,457,576	—	7 (low)
Mulla	2013	United States	Cross-sectional	Non-probabilistic	Deliveries in Florida/Texas with preeclampsia and GBS data	767,798	26	7 (low)

Park	2013	Australia	Cohort	Non-probabilistic	First trimester screening (11-13+6 weeks); excluded: multiple pregnancies	3,099	—	7 (low)
Xiao	2014	China	Cross-sectional	Probabilistic	Gestation >20 weeks; excluded: comorbidities and IVF	67,746	26	8 (low)
Baragou	2014	Togo	Cross-sectional	Probabilistic	Pregnant women; excluded: transitory hypertension during delivery	1,620	30	8 (low)
Torjusen	2014	Norway	Cohort	Probabilistic	Nulliparous with complete data; excluded: multiple pregnancies	28,192	29	8 (low)
Oliveira	2014	United States	Cross-sectional	Probabilistic	First trimester screening; excluded: aspirin use	2,446	—	8 (low)
Tessema	2015	Ethiopia	Cohort	Probabilistic	Pregnant women ≥20 weeks; excluded: unreliable gestational age	490	27	8 (low)
Vata	2015	Ethiopia	Cohort	Non-probabilistic	First trimester evaluation; excluded: previous aspirin use	7,702	33	7 (low)
Park F	2015	Australia	Cross-sectional	Probabilistic	Prenatal consultation in Tizi-ouzou (2012-2013); excluded: chronic hypertension	5,783	30.2	8 (low)
Naimy	2015	Norway	Cross-sectional	Probabilistic	Deliveries >20 weeks of Norwegian women and migrants	1,102,189	—	8 (low)
Kichou	2015	Algeria	Cross-sectional	Probabilistic	Diagnosis of preeclampsia/eclampsia according to ICD-9-CM	3,225	—	8 (low)
Cho	2016	South Korea	Cohort	Probabilistic	Primiparous women (2011-2012); excluded: preexisting hypertension	212,463	30.44	8 (low)
Marchand	2016	Mongolia	Cross-sectional	Probabilistic	Pregnant women in Mongolia without initial complications	221	—	8 (low)
Rezende	2016	Brazil	Cross-sectional	Non-probabilistic	Singleton pregnancy at UFRJ, delivery ≥22 weeks (2011-2012)	4,464	—	7 (low)
Yamada	2016	Japan	Cross-sectional	Non-probabilistic	Singleton pregnancies in Japan with protein/creatinine measurement	6,819	—	7 (low)

Labarca	2017	Venezuela	Retrospective cohort	Non-probabilistic	Critical pregnant women admitted to ICU, Armando Castillo Plaza Maternity, Maracaibo (2011-2015). HELLP syndrome diagnosis.	816	25.8	5 (moderate)
Cremonte	2017	Argentina	Retrospective cohort	Non-probabilistic	Women with HELLP syndrome, Hospital Ángela Iglesias Llano, Corrientes (2015-2017). Age 15-45 years, increased BMI prior to pregnancy or previous hypertension.	580	28.1	8 (low)
Auger	2017	Canada	Cohort	Probabilistic	Deliveries Quebec (1989-2013), gestation >20 weeks	606,820	—	8 (low)
Bellizzi	2017	Multi-country (Colombia, Bangladesh, Indonesia, Mali, Niger, Peru)	Cross-sectional	Non-probabilistic	Singleton deliveries (2005-2012).	55,384	Not specified overall	6 (moderate)
Nehbandani	2018	Iran	Cross-sectional	Non-probabilistic	Pregnant women in emergency area of Hospital Verdi Cevallos (2016-2017), pregnancy ≥20 weeks, complete clinical diagnosis	2,000		5 (moderate)
Mahran	2017	Egypt	Cross-sectional	Non-probabilistic	Confirmed eclampsia; excluded: other causes of seizures	21,690	—	6 (moderate)
Reichelt	2017	Brazil	Retrospective cohort	Non-probabilistic	Women with gestational diabetes treated at university hospitals (Porto Alegre, 1991-1993 and 2009-2013)	375	31	7 (low)
Sucksdorf	2017	Argentina	Prospective cohort	Non-probabilistic	Pregnant women admitted to Sanatorio San Gerónimo, Santa Fe, Argentina (3 months, 2016)	136	Not specified overall	7 (low)

Rana	2018	Nepal	Retrospective cohort	Non-probabilistic	Women with pregnancies ≥20 weeks treated at Amiralmomenin hospital, Zabol (2014-2015).	16,445		7 (low)
Hutcheon	2018	Sweden	Cohort	Probabilistic	Nulliparous women (2008-2013) with BMI data; excluded: previous hypertension	62,705	—	8 (low)
Di Martino	2019	Italy	Cross-sectional	Non-probabilistic	Pregnant women treated (2011-2016) National Hospital, Birgunj, Nepal, confirmed diagnosis of eclampsia during gestation, delivery, or immediate postpartum.	11,632		7 (low)
Leon	2019	United Kingdom	Cohort	Probabilistic	Pregnancies (1997-2016); excluded: <20 weeks, incomplete records	1,303,365	28	8 (low)
Laine	2019	Norway	Cohort	Probabilistic	Singleton/twin pregnancies (1999-2014); excluded: deliveries <22 weeks	929,963	—	8 (low)
Sanchéz	2019	Colombia	Cross-sectional	Non-probabilistic	Pregnant women treated at Health Institution, Boyacá, 2015-2017. Age ≥20 weeks, clinically confirmed diagnosis.	2,531		7 (low)
Liu	2019	China	Cohort	Probabilistic	Singleton pregnancies (1993-1995); excluded: comorbidities, anomalies	199,231	24.6	8 (low)
Mayrink	2019	Brazil	Cohort	Probabilistic	Nulliparous women in Brazil without chronic diseases	1,165	—	8 (low)
Nurgaliweva	2020	Kazakhstan	Cross-sectional	Probabilistic	Preeclampsia in Kazakhstan (2017); excluded: incomplete records	2,548	28	8 (low)
Utami	2020	Indonesia	Cross-sectional	Non-probabilistic	Women with pregnancy ≥20 weeks in Sidoarjo Regency, Indonesia, between 2015 and 2019.	179,609	Not specified overall	7 (low)

Sitaula	2020	Nepal	Cross-sectional	Non-probabilistic	Pregnant women with HELLP syndrome treated at tertiary hospital, Nepal (April 2017-March 2018).	11,974	24.8	7 (low)
Filho	2020	Brazil	Retrospective cohort	Non-probabilistic	Women hospitalized for delivery, Maternidade Hilda Brandão, Belo Horizonte (2008-2018)	36,724	Not specified overall	7 (low)
Abu-Zaid	2020	Saudi Arabia	Cross-sectional	Probabilistic	Preeclampsia (2011-2018), age ≥18; excluded: multiple pregnancies	3,942	—	8 (low)
Rorman	2020	Israel	Cohort	Probabilistic	Births (1991-2014); excluded: malformations, perinatal deaths	239,725	—	8 (low)
Corrigan	2021	Ireland	Cross-sectional	Probabilistic	Hypertensive disorder in Ireland (2016); excluded: previous hypertension	60,188	—	8 (low)
Ybaseta-Medina	2021	Peru	Cross-sectional	Non-probabilistic	Pregnant women treated with gestational hypertensive disorders between 2017-2019 at Hospital Santa María del Socorro, Ica, Peru. No explicit exclusion for previous comorbidities.	246		7 (low)
Trindade	2021	Brazil	Prospective cohort	Non-probabilistic	Healthy nulliparous pregnant women, without previous hypertension, singleton pregnancy, weeks 17-20, excluding pregestational diabetes, cardiac or renal diseases	196	25.1	7 (low)
Sanchez	2021	Brazil	Cross-sectional	Non-probabilistic	Women treated at university hospital Campinas, São Paulo, January 2017 to February 2018	3,102	28.7	7 (low)
Olié	2021	France	Cohort	Probabilistic	Deliveries in France (2010-2018); excluded: anonymous deliveries	6,302,810	—	8 (low)

Yang	2021	Sweden and China	Cross-sectional	Probabilistic	Pregnancies ≥22 weeks; excluded: previous hypertension	Sweden: 555,446<br>China: 79,243	Sweden: 30.9<br>China: 28.6	8 (low)
Rezende	2021	Brazil	Cross-sectional	Non-probabilistic	First trimester screening; excluded: chromosomal anomalies	1,695	—	7 (low)
Akaba	2021	Nigeria	Cross-sectional	Non-probabilistic	Pregnant women treated between 2014-2019, Abuja University Hospital, Nigeria, without prior chronic hypertension or before week 20	9,760	30.1	7 (low)
Tejera	2021	Ecuador	Retrospective cohort	Non-probabilistic	All deliveries registered in Ecuador (2015-2017), including women of different ethnicities, located from sea level to altitudes >3500 m.a.s.l. Parity and socioeconomic status not available.	494,045		7 (low)
Boakye	2021	United States	Cross-sectional	Non-probabilistic	Non-Hispanic Black women with preeclampsia data	2,697	—	7 (low)
Chamyan	2021	Uruguay	Retrospective cohort	Non-probabilistic	Pregnant women with deliveries between 2014-2018 at Hospital Clínicas Dr. Manuel Quintela, Uruguay, with confirmed preeclampsia-eclampsia syndrome.	3,590	Not specified	7 (low)
Sutan	2022	Malaysia	Cross-sectional	Non-probabilistic	Deliveries ≥20 weeks, Hospital Tunku Muhriz (2010-2020)	40,212	—	7 (low)
Huang	2022	Denmark	Cohort	Probabilistic	Live births Denmark (1978-2018); excluded: <22 weeks	2,437,718	19.4	8 (low)
Paudyal	2022	Nepal	Cross-sectional	Non-probabilistic	Preeclampsia with HELLP criteria; excluded: previous pathologies	19,163	28.05	7 (low)

An	2022	China	Cohort	Probabilistic	Pregnant women in China (1993-1995); excluded: previous hypertension	200,103	—	8 (low)
Wheeler	2022	USA	Retrospective cohort	Non-probabilistic	All births registered in USA in 2019, excluded undocumented risk factors (previous history of preeclampsia, renal disease, autoimmune).	3,695,019	29.1	7 (low)
Anjum	2024	India	Cross-sectional	Probabilistic	Pregnant women 18-24 weeks; excluded: comorbidities, initial proteinuria	100	23.53	8 (low)
Shandilya	2024	India	Cross-sectional	Probabilistic	Pregnant women >20 weeks; excluded: eclampsia, gestational hypertension	500	28.7	8 (low)
Sun	2024	China	Cohort	Probabilistic	Singleton pregnancies with complete follow-up; excluded: previous pathologies	34,104	31.12	8 (low)
Adjie	2023	Indonesia	Cross-sectional	Non-probabilistic	Severe preeclampsia with risk factors; excluded: <20 weeks	839	—	7 (low)
Vásquez	2023	Spain	Cohort	Non-probabilistic	Pregnant women in first trimester; excluded: <18 years, pathologies	124	34.2	7 (low)
Anto	2024	Ghana	Cross-sectional	Probabilistic	Pregnant women 16-45 years, ≥20 weeks; excluded: multiple gestations	1,174	—	8 (low)
Awor	2024	Uganda	Cohort	Probabilistic	Pregnant women 16-24 weeks; excluded: previous pathologies, fetal death	1,004	—	8 (low)
Swiercz	2024	Poland	Cross-sectional	Probabilistic	Women with first trimester tests (2018-2019)	1,212	30.2	8 (low)
Riishede	2024	Denmark	Cohort	Probabilistic	Singleton pregnancies in first trimester; excluded: <18 years, language barrier	8,386	30.8	8 (low)

Humayun	2024	Pakistan	Cross-sectional	Non-probabilistic	Confirmed eclampsia; excluded: epilepsy, metabolic causes	4,561	—	7 (low)
Sakthi	2024	India	Cross-sectional	Non-probabilistic	Eclampsia up to 42 days postpartum; excluded: previous epilepsy	15,117	—	7 (low)
Lailler	2024	France	Cohort	Probabilistic	Primiparous women (2010-2018); excluded: <15 or >49 years, previous diabetes	2,816,793	28.8	8 (low)
Rashid	2024	Tanzania	Cross-sectional	Probabilistic	Women ≥20 weeks with consent	138	38.96	8 (low)
Chai	2025	China	Retrospective cohort	Non-probabilistic	Pregnant women between 18 to 55 years, gestation ≥22 weeks, without pregestational diabetes, chronic hypertension, cardiac or renal disease; triple pregnancies and fetal chromosomal abnormalities were excluded	39,626	31.96	7 (low)

Supplementary Material 3. Meta-analysis of the global prevalence of preeclampsia

Study	Country	Cases	Total	Proportion	95%-CI	Weight
**Criteria = ACOG**						
Gaio 2001	Brazil	113	4892	0.023	[0.019; 0.028]	1.5%
Gonçalves 2005	Brazil	22	604	0.036	[0.023; 0.055]	1.3%
Aliyu 2010	United States	14540	290807	0.050	[0.049; 0.051]	1.5%
Oliveira N 2014	United States	20	2446	0.008	[0.005; 0.013]	1.5%
Xiao J 2014	China	1597	67746	0.024	[0.022; 0.025]	1.5%
Tessema GA 2015	Ethiopia	41	490	0.084	[0.061; 0.112]	1.3%
Naimy Z 2015	Norway	39376	1102189	0.036	[0.035; 0.036]	1.5%
Kichou B 2015	Algeria	252	3225	0.078	[0.069; 0.088]	1.5%
Cho GJ 2016	South Korea	6585	212463	0.031	[0.030; 0.032]	1.5%
Marchand NE 2016	Mongolia	9	221	0.041	[0.021; 0.078]	1.1%
Rezende KBC 2016	Brazil	301	4464	0.067	[0.060; 0.075]	1.5%
Cremonte 2017	Argentina	20	580	0.034	[0.022; 0.053]	1.3%
Bellizzi 2017	Multicountry (Colombia, Bangladesh, Indonesia, Mali, Niger, Peru)	665	55384	0.012	[0.011; 0.013]	1.5%
Labarca 2017	Venezuela	75	816	0.092	[0.073; 0.114]	1.4%
Reichelt 2017	Brazil	53	375	0.141	[0.108; 0.181]	1.2%
Milos 2017	Argentina	3	136	0.022	[0.008; 0.066]	0.9%
Salamanca 2019	Colombia	90	2531	0.036	[0.029; 0.044]	1.5%
Rorman E 2020	Israel	7280	239725	0.030	[0.030; 0.031]	1.5%
Sanchez 2020	Brazil	258	3102	0.083	[0.074; 0.094]	1.5%
Ramos Filho 2020	Brazil	2531	36724	0.069	[0.066; 0.072]	1.5%
Chamyan 2021	Uruguay	286	3590	0.080	[0.071; 0.089]	1.5%
Medina 2021	Peru	32	246	0.130	[0.091; 0.179]	1.1%
Trindade 2021	Brazil	12	196	0.061	[0.034; 0.106]	1.0%
An H 2022	China	4912	200103	0.025	[0.024; 0.025]	1.5%
Huang C 2022	Denmark	67683	2437718	0.028	[0.027; 0.027]	1.5%
Vásquez S 2023	Spain	8	124	0.065	[0.032; 0.126]	0.9%
Adjie JM 2023	Indonesia	109	839	0.130	[0.108; 0.155]	1.4%
Anjum M 2024	India	14	100	0.140	[0.082; 0.226]	0.8%
Chai 2025	China	1130	39626	0.029	[0.026; 0.029]	1.5%
**Overall Random-Effects**			4711462	0.046	[0.035; 0.059]	39.4%
Heterogeneity: $I^2 = 100\%$, $p = 0$						
**Criteria = ISSHP**						
Immink 2008	South Africa	1079	11585	0.093	[0.085; 0.096]	1.5%
Direkvand-Moghadam 2013	Iran	58	610	0.095	[0.074; 0.122]	1.3%
Shiozaki 2013	Japan	6423	241292	0.027	[0.026; 0.027]	1.5%
Von Schmicdt 2013	Netherlands	31560	1457576	0.022	[0.021; 0.022]	1.5%
Park FJ 2013	Australia	71	3099	0.023	[0.018; 0.029]	1.5%
Baragou S 2014	Togo	88	1620	0.054	[0.044; 0.067]	1.4%
Park F 2015	Australia	12	5783	0.002	[0.001; 0.004]	1.5%
Vata pk 2015	Ethiopia	172	7702	0.022	[0.019; 0.026]	1.5%
Yamada T 2016	Japan	158	6819	0.023	[0.020; 0.027]	1.5%
Handayani 2017	Indonesia	10310	179609	0.057	[0.056; 0.058]	1.5%
Hutcheon JA 2018	Sweden	1920	62705	0.031	[0.029; 0.032]	1.5%
Nehbandani 2018	Iran	130	2000	0.065	[0.055; 0.077]	1.5%
Di Martino D 2019	Italy	278	11632	0.024	[0.021; 0.027]	1.5%
Laine K 2019	Norway	34150	929963	0.037	[0.036; 0.037]	1.5%
Leon LJ 2019	United Kingdom	31478	1303365	0.024	[0.024; 0.024]	1.5%
Liu L 2019	China	4906	199231	0.025	[0.024; 0.025]	1.5%
Mayirink J 2019	Brazil	87	1165	0.075	[0.061; 0.092]	1.4%
Nurgaliweva GT 2020	Kazakhstan	54	2548	0.021	[0.016; 0.028]	1.5%
Abu-Zaid A 2020	Saudi Arabia	167	3942	0.042	[0.036; 0.049]	1.5%
Yang Y - 1 2021	Sweden	16068	555446	0.029	[0.028; 0.029]	1.5%
Yang Y - 2 2021	China	1803	79243	0.023	[0.022; 0.024]	1.5%
Corrigan L 2021	Ireland	2790	60188	0.046	[0.043; 0.046]	1.5%
Rezende KBC 2021	Brazil	164	1695	0.097	[0.083; 0.112]	1.4%
Tejera 2021	Ecuador	15390	494045	0.031	[0.028; 0.029]	1.5%
Olié 2021	France	126056	6302810	0.020	[0.020; 0.020]	1.5%
Okaba 2021	Nigeria	295	9760	0.030	[0.027; 0.034]	1.5%
Sutan R 2022	Malaysia	641	40212	0.016	[0.015; 0.017]	1.5%
Wheeler 2022	United States	300080	3695019	0.081	[0.081; 0.081]	1.5%
Shandilya V 2024	India	31	500	0.062	[0.043; 0.088]	1.3%
Sun M 2024	China	788	34104	0.023	[0.018; 0.021]	1.5%
Anto EO 2024	Ghana	103	1174	0.088	[0.072; 0.105]	1.4%
Awor S 2024	Uganda	43	1004	0.043	[0.031; 0.057]	1.4%
Swiercz G 2024	Poland	17	1212	0.014	[0.009; 0.023]	1.4%
Riishede I 2024	Denmark	313	8386	0.037	[0.029; 0.037]	1.5%
**Overall Random-Effects**			15717044	0.037	[0.029; 0.046]	50.6%
Heterogeneity: $I^2 = 100\%$, $p = 0$						
**Criteria = National High Blood Pressure Education Program**						
Wendland 2008	Brazil	148	4766	0.031	[0.026; 0.036]	1.5%
Boakye E 2021	United States	299	2697	0.111	[0.099; 0.123]	1.5%
**Overall Random-Effects**			7463	0.065	[0.000; 0.923]	3.0%
Heterogeneity: $I^2 = 99\%$, $p < 0.01$						
**Criteria = ICD-9**						
Mulla ZD 2013	United States	29398	767798	0.038	[0.038; 0.039]	1.5%
**Criteria = Norwegian Medical Birth Registry**						
Torjusen H 2014	Norway	1491	28192	0.053	[0.050; 0.055]	1.5%
**Criteria = ICD-10**						
Auger N 2017	Canada	24799	606820	0.041	[0.037; 0.038]	1.5%
Lailler G 2024	France	81258	2816793	0.029	[0.029; 0.029]	1.5%
**Overall Random-Effects**			3423613	0.035	[0.000; 0.149]	3.1%
Heterogeneity: $I^2 = 100\%$, $p = 0$						
**Criteria = OMS**						
Rashid SA 2024	Tanzania	20	138	0.145	[0.094; 0.217]	0.9%
**Overall Random-Effects**			24655710	0.044	[0.037; 0.051]	100.0%
Heterogeneity: $I^2 = 100\%$, $p = 0$						
Test for subgroup differences: $\chi^2_6 = 158.69$, df = 6 ($p < 0.01$)						

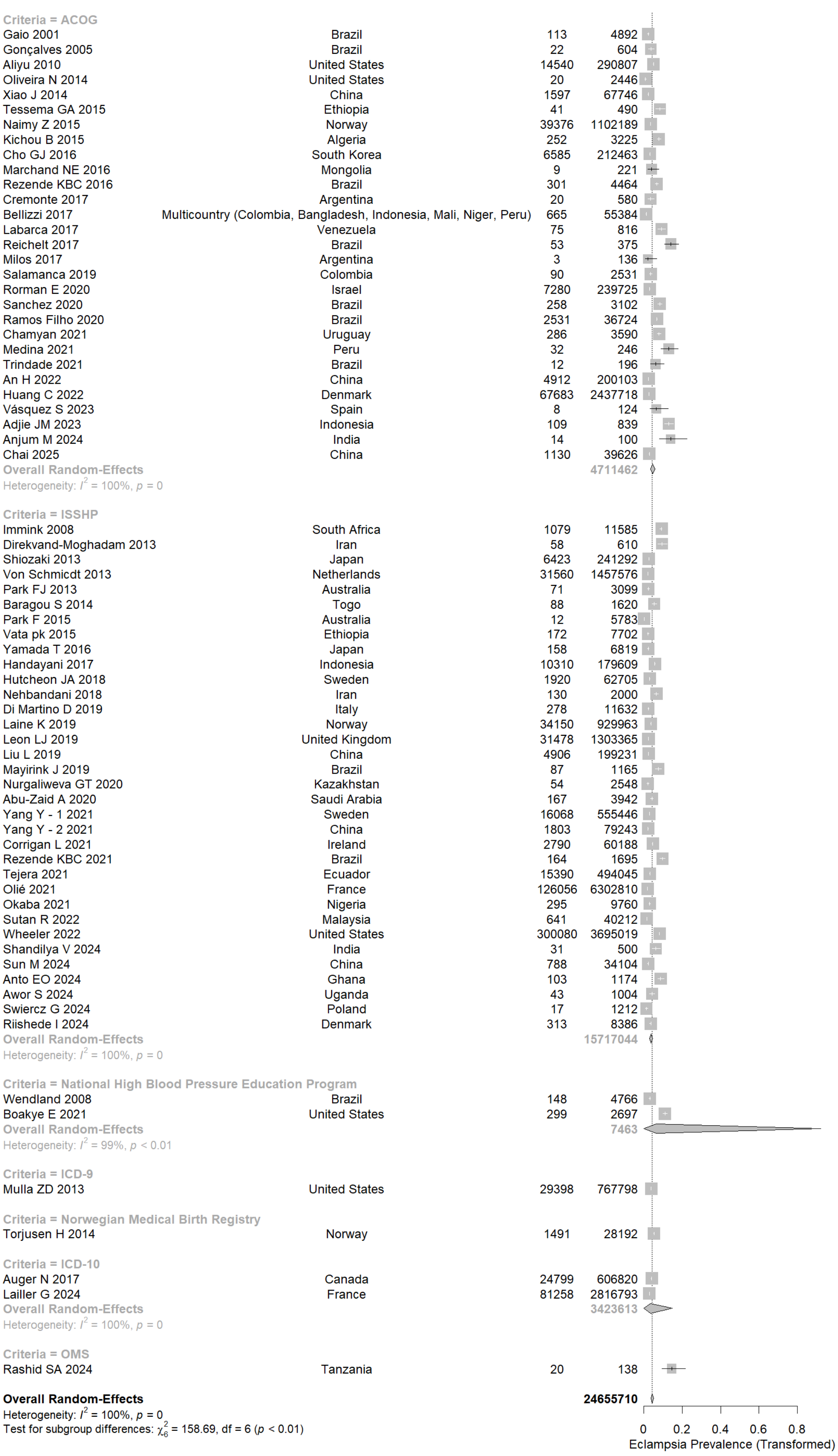

Abbreviations: ACOG, American College of Obstetricians and Gynecologists; ISSHP, International Society for the Study of Hypertension in Pregnancy; ICD-10, International Classification of Diseases, 10th Revision; CI, Confidence Interval; I², I-squared statistic for heterogeneity; df, degrees of freedom; χ², Chi-squared test statistic.

Supplementary Material 4. Meta-analysis of the global prevalence of eclampsia

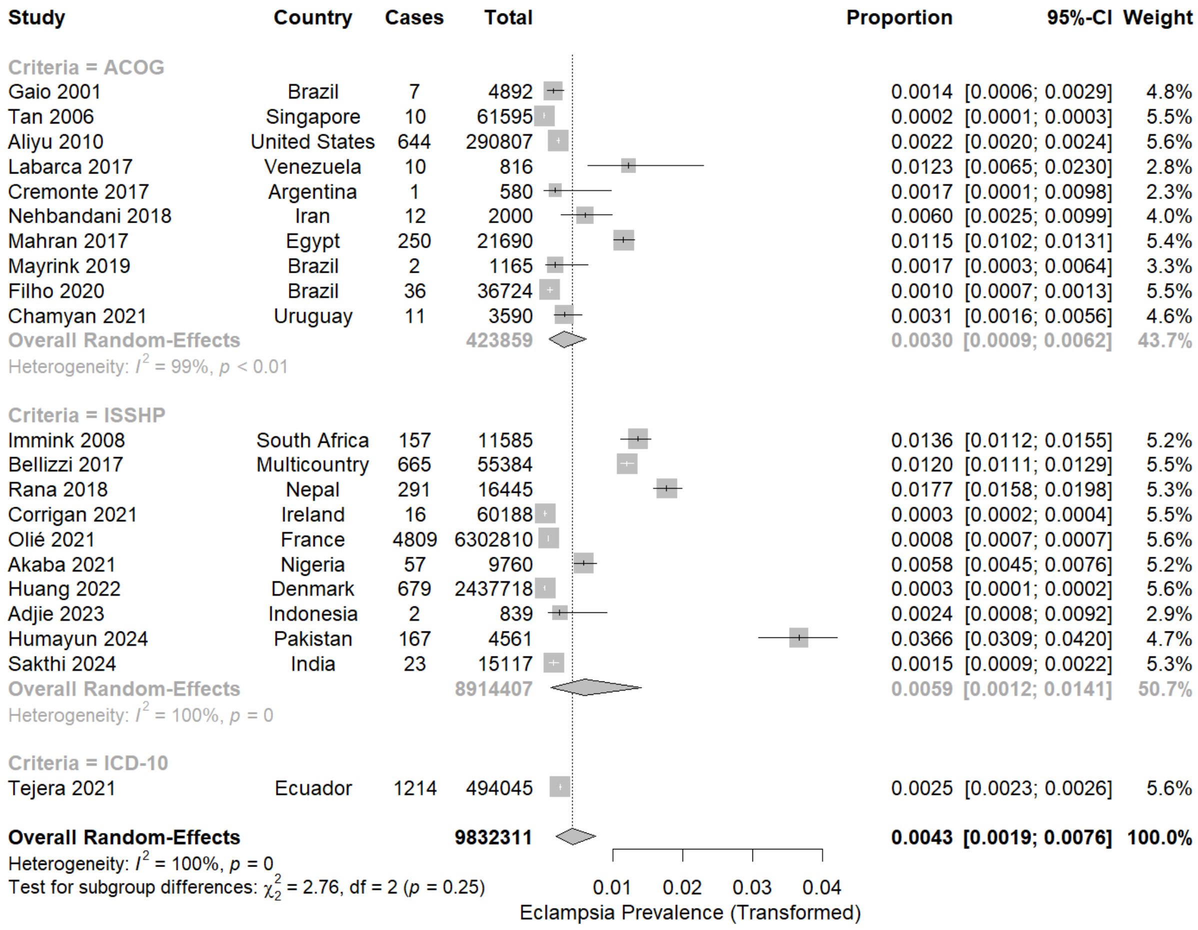

Abbreviations: ACOG, American College of Obstetricians and Gynecologists; ISSHP, International Society for the Study of Hypertension in Pregnancy; ICD-10, International Classification of Diseases, 10th Revision; CI, Confidence Interval; I², I-squared statistic for heterogeneity; df, degrees of freedom; χ², Chi-squared test statistic.

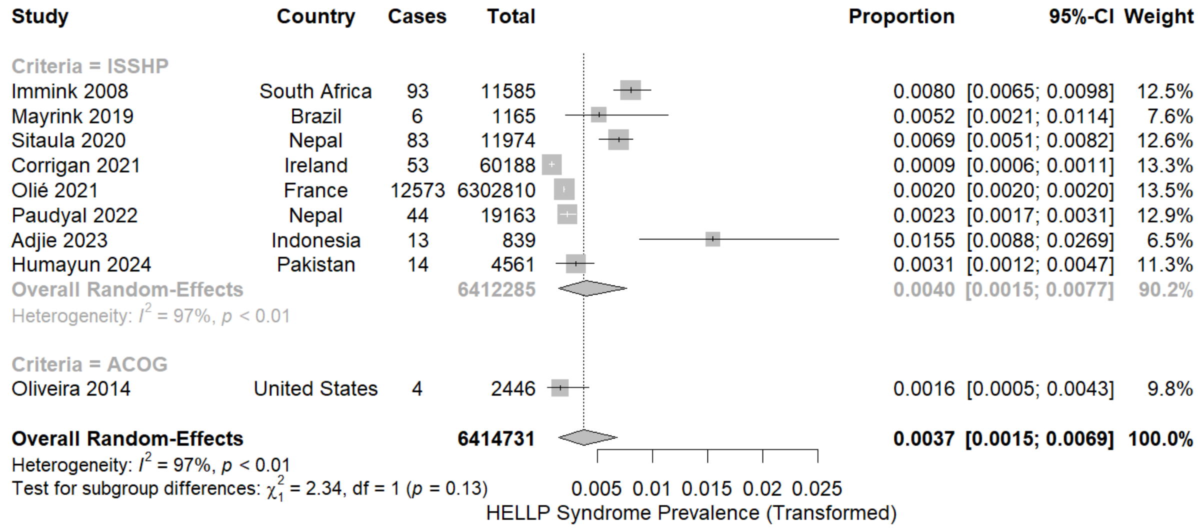

Supplementary Material 5. Meta-analysis of the global prevalence of HELLP syndrome

Abbreviations: ACOG, American College of Obstetricians and Gynecologists; ISSHP, International Society for the Study of Hypertension in Pregnancy; ICD-10, International Classification of Diseases, 10th Revision; CI,

Confidence Interval; I^2, I-squared statistic for heterogeneity; df, degrees of freedom; χ^2, Chi-squared test statistic.

Supplementary Material 6. Global prevalence of preeclampsia, eclampsia and Hellp syndrome by country

Country	Preeclampsia		Eclampsia		Hellp Syndrome	
	Number of Studies	Prevalence (%) (IC 95%)	Number of Studies	Prevalence (%) (IC 95%)	Number of Studies	Prevalence (%) (IC 95%)
Algeria	1	7.81 (6.91–8.79)	—	—	—	—
Argentina	1	3.21 (2.05–4.78)	1	0.17 (0.00–0.74)	—	—
Australia	1	0.93 (0.74–1.16)	—	—	—	—
Brazil	1	6.36 (6.16–6.56)	3	0.09 (0.04–0.17)	1	0.52 (0.17–1.02)
Canada	1	4.09 (4.04–4.14)	—	—	—	—
China	1	2.44 (2.40–2.48)	—	—	—	—
Colombia	1	3.56 (2.87–4.35)	—	—	—	—
Denmark	1	2.78 (2.76–2.80)	1	0.03 (0.03–0.03)	—	—
Ecuador	1	3.12 (3.07–3.16)	1	0.25 (0.23–0.26)	—	—
Egypt	—	—	1	1.15 (1.01–1.30)	1	0.18 (0.13–0.24)
Ethiopia	1	2.60 (2.27–2.97)	—	—	—	—
France	1	2.27 (2.26–2.28)	1	0.08 (0.07–0.08)	1	0.20 (0.20–0.20)
Ghana	1	8.77 (7.22–10.54)	—	—	—	—
India	1	7.50 (5.52–9.91)	1	0.15 (0.10–0.22)	—	—
Indonesia	1	5.77 (5.67–5.88)	1	0.24 (0.00–0.72)	1	1.55 (0.81–2.51)
Iran	1	7.20 (6.24–8.26)	1	0.60 (0.30–0.99)	—	—
Ireland	1	4.64 (4.47–4.81)	1	0.03 (0.01–0.04)	1	0.09 (0.07–0.11)
Israel	1	3.04 (2.97–3.11)	—	—	—	—
Italy	1	2.39 (2.12–2.68)	—	—	—	—
Japan	1	2.65 (2.59–2.72)	—	—	—	—
Kazakhstan	1	2.12 (1.60–2.76)	—	—	—	—
Madagascar	—	—	1	1.51 (1.16–1.89)	—	—
Malaysia	1	1.59 (1.47–1.72)	—	—	—	—
Mongolia	1	4.07 (1.88–7.59)	—	—	—	—
Multicountry	1	1.20 (1.11–1.29)	1	1.20 (1.11–1.29)	—	—
Nepal	—	—	1	1.77 (1.57–1.98)	2	0.43 (0.00–8.21)
Netherlands	1	2.17 (2.14–2.19)	—	—	—	—

Nigeria	1	3.02 (2.69–3.38)	1	0.58 (0.44–0.75)	—	—
Norway	1	3.64 (3.62–3.67)	—	—	—	—
Pakistan	—	—	2	2.02 (0.00–48.84)	1	0.31 (0.16–0.49)
Peru	1	13.01 (9.07–17.86)	—	—	—	—
Poland	1	1.40 (0.82–2.24)	—	—	—	—
Saudi Arabia	1	4.24 (3.63–4.91)	—	—	—	—
Singapore	—	—	1	0.02 (0.01–0.03)	—	—
South Africa	1	9.31 (8.79–9.86)	1	1.36 (1.15–1.57)	1	0.80 (0.65–0.97)
South Korea	1	3.10 (3.03–3.17)	—	—	—	—
Spain	1	6.45 (2.83–12.32)	—	—	—	—
Sweden	1	2.91 (2.87–2.95)	—	—	—	—
Switzerland	—	—	1	0.31 (0.07–0.70)	1	0.23 (0.03–0.58)
Taiwan	—	—	1	0.03 (0.03–0.03)	—	—
Tanzania	1	14.49 (9.08–21.49)	—	—	—	—
Togo	1	5.43 (4.38–6.65)	—	—	—	—
Uganda	1	4.28 (3.12–5.73)	—	—	—	—
United Kingdom	1	2.42 (2.39–2.44)	—	—	—	—
United States	1	7.24 (7.21–7.26)	1	0.22 (0.20–0.24)	1	0.16 (0.03–0.37)
Uruguay	1	7.97 (7.10–8.90)	1	0.31 (0.15–0.52)	—	—
Venezuela	1	9.19 (7.30–11.38)	1	1.23 (0.57–2.11)	—	—

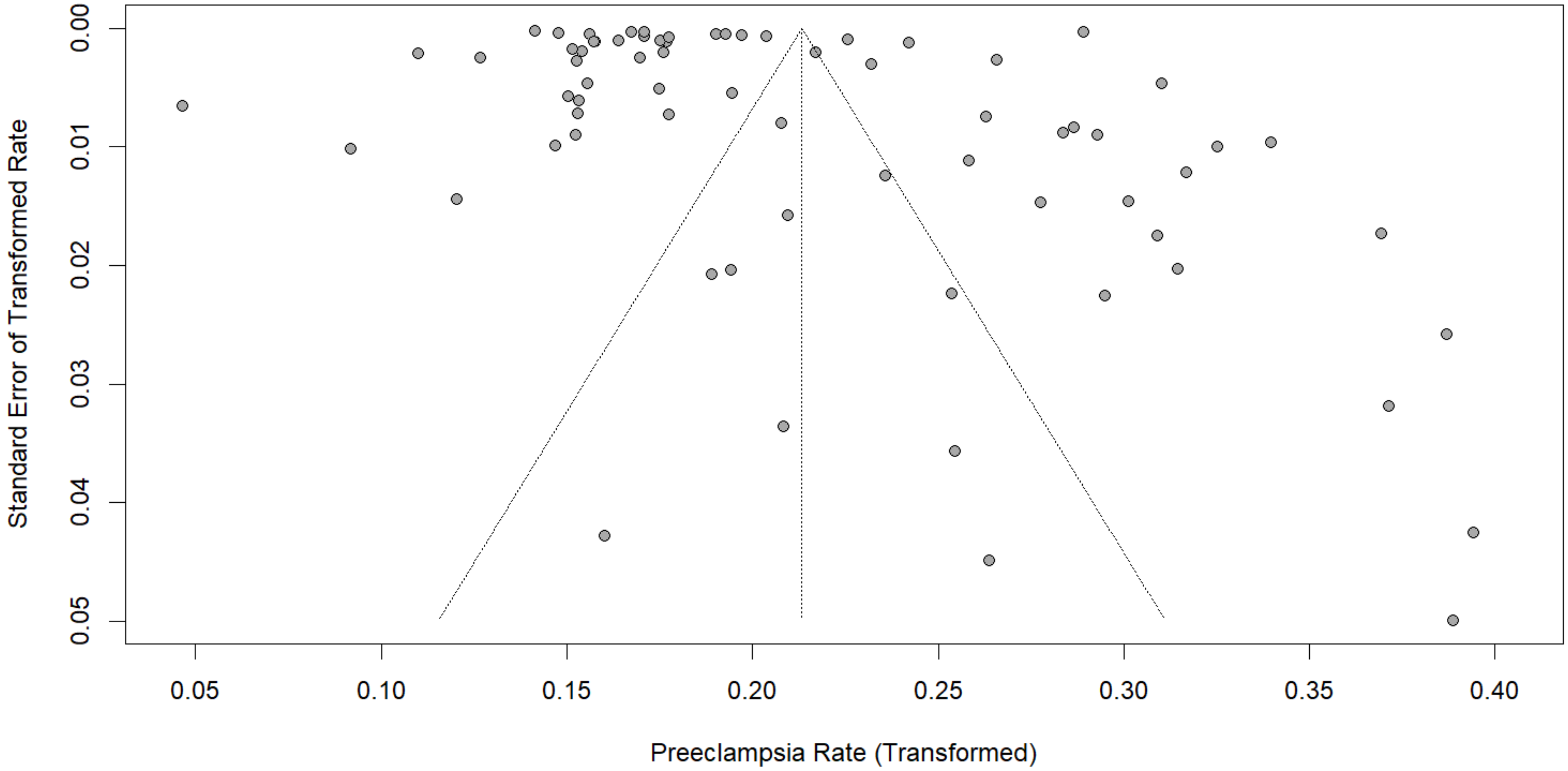

Supplementary Material 7. Funnel plot (Freeman-Tukey arcsine transformation) of the prevalence of preeclampsia

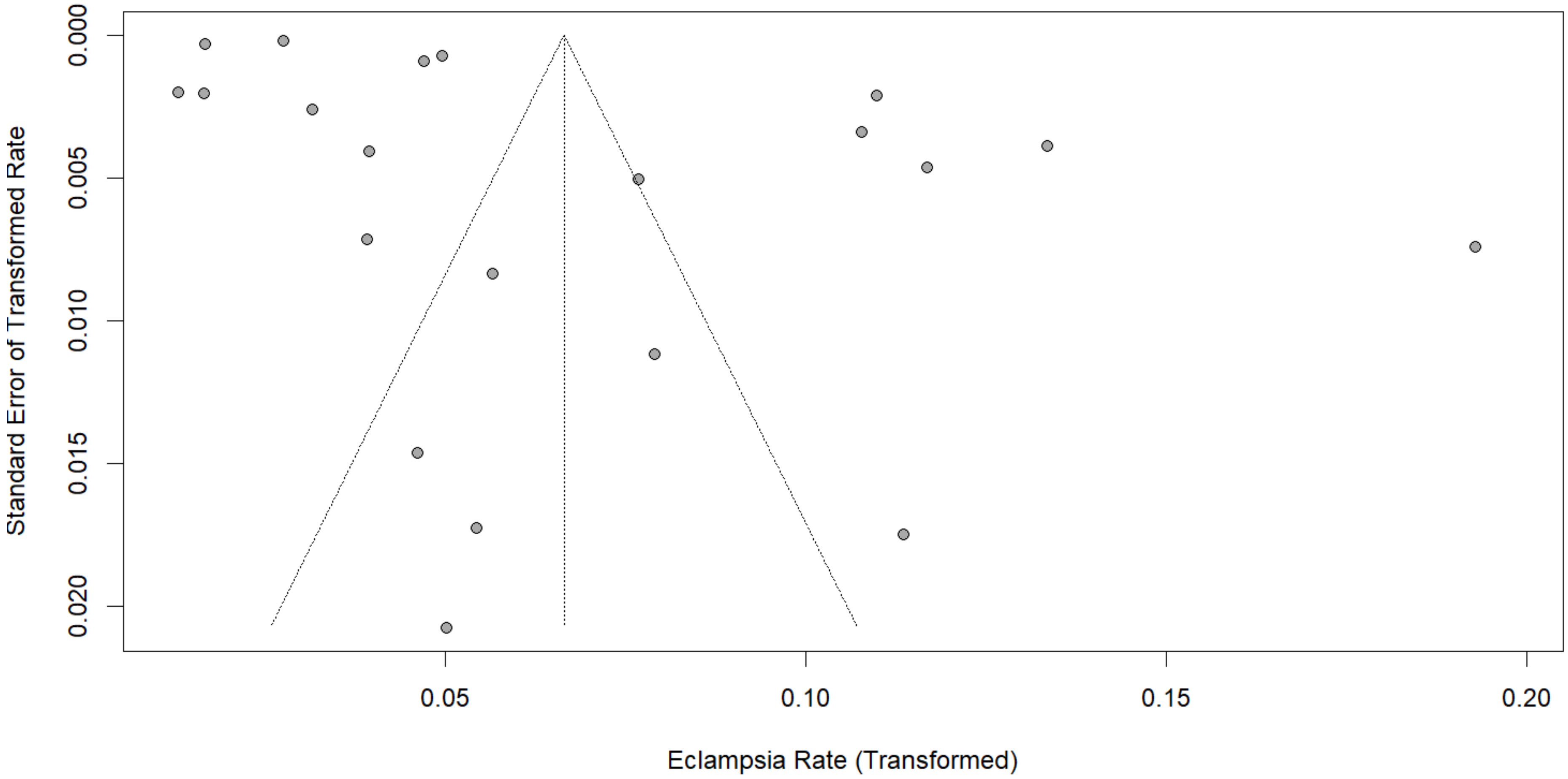

Supplementary Material 8. Funnel plot (Freeman-Tukey arcsine transformation) of the prevalence of eclampsia

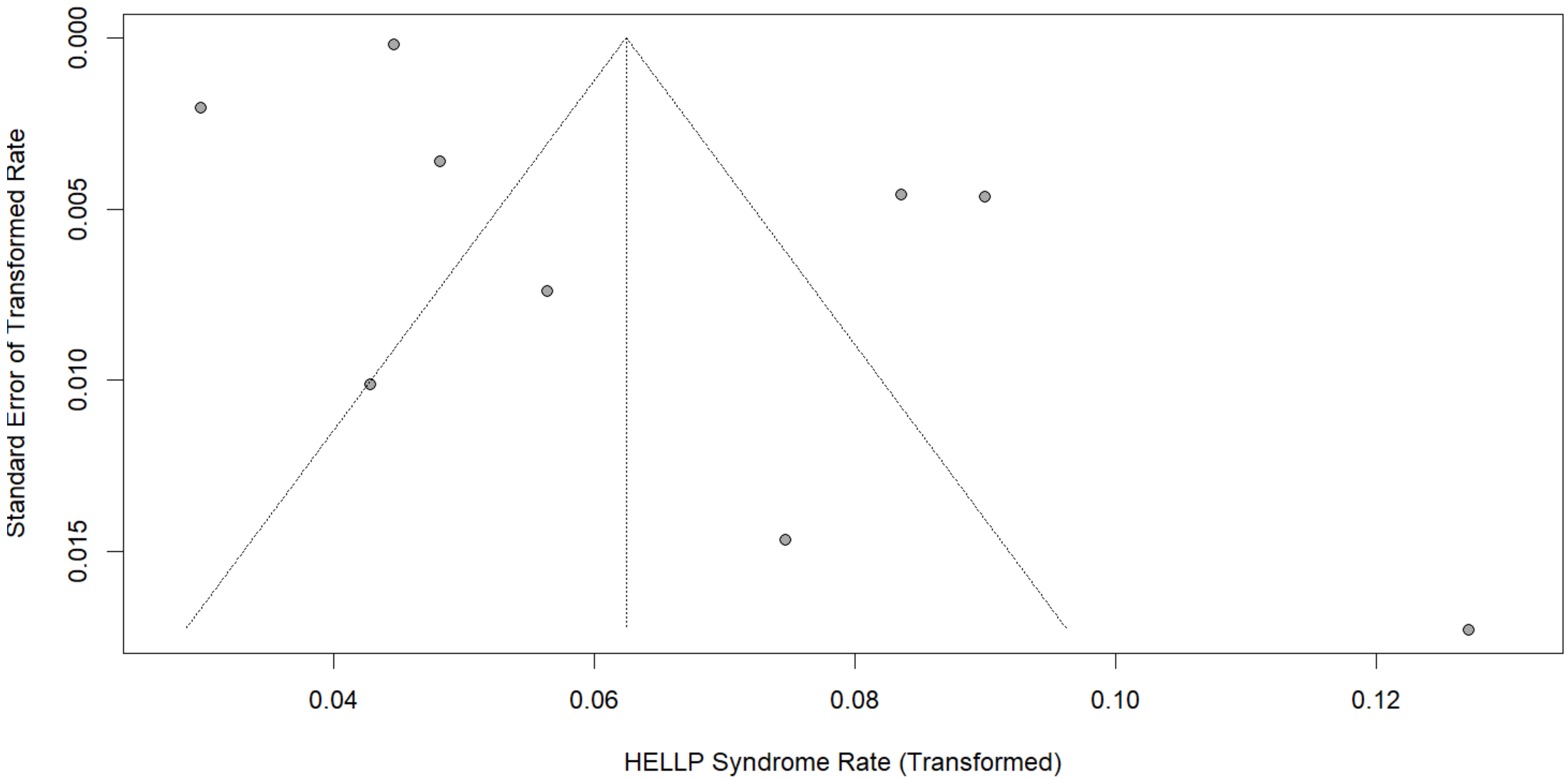

Supplementary Material 9. Funnel plot (Freeman-Tukey arcsine transformation) of the prevalence of HELLP syndrome

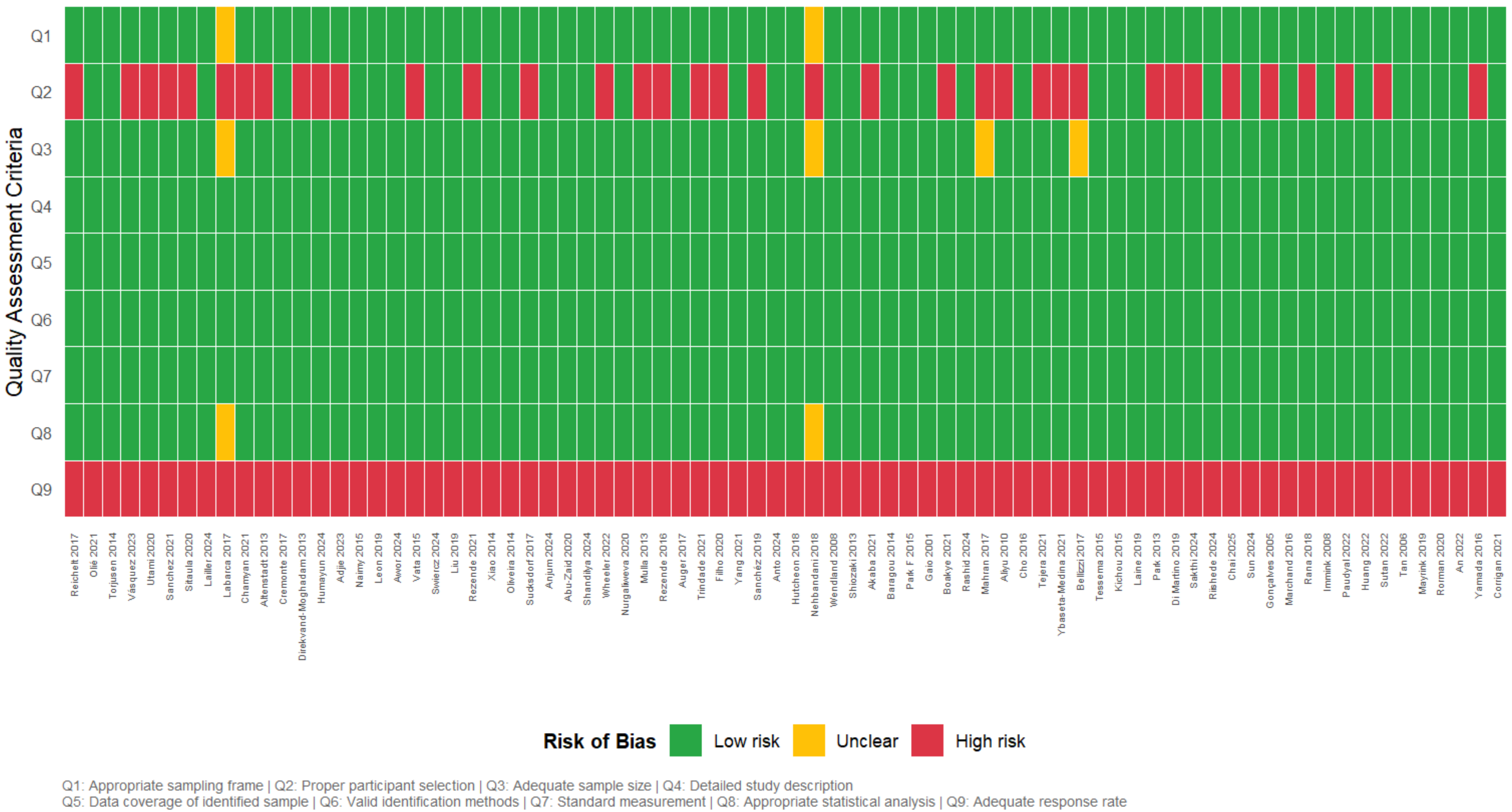

Supplementary Material 10. Risk of bias assessment
